# Supplementary material for: Systematic review of the evidence on orthotic devices for the management of knee instability related to neuromuscular and central nervous system disorders
Source: BMJ Open. 2017 Sep 5;7(9):e015927. doi: 10.1136/bmjopen-2017-015927 (PMC5588970; doi:10.1136/bmjopen-2017-015927)
Supplement: Supplementary file 1 [file bmjopen-2017-015927supp001.pdf]

SUPPLEMENTARY APPENDIX 1

Database: Ovid MEDLINE(R) In-Process & Other Non-Indexed Citations and Ovid MEDLINE(R) <1946 to Present> (searched online 22/05/14)

Search Strategy:

1 Orthotic Devices/ or Braces/ or Splints/ (16320)

2 Gait/ (17744)

3 Lower Extremity/ or Leg/ (61929)

4 Hip/ or Hip Joint/ (28943)

5 Knee/ or exp Knee Joint/ (51355)

6 Ankle/ or Ankle Joint/ (16707)

7 Foot/ or Foot Joints/ (20388)

8 1 and (2 or 3 or 4 or 5 or 6 or 7) (2732)

9 Foot Orthoses/ (145)

10 8 or 9 (2870)

11 ((gait or "lower extremity" or "lower extremities" or "lower limb" or "lower limbs" or leg? or hip? or knee? or ankle? or foot or feet) adj3 (orthos* or orthot* or brace? or bracing or support)).ti,ab. (3590)

12 (heel adj2 (pad? or raise?)).ti,ab. (365)

13 ((shoe? and (modification? or insert? or "negative heel" or "negative heels")) or (rocker? or insole?)).ti,ab. (1507)

14 ((HKAFO? or KAFO? or SCKAFO? or AFO? or GRAFO? or RGO? or SWASH? or DAFO? or SAFO?) and (orthos* or orthot* or brace? or bracing)).ti,ab. (387)

15 (SMART? and walker).ti,ab. (10)

16 11 or 12 or 13 or 14 or 15 (5269)

17 10 or 16 (6735)

18 exp Knee Joint/ or Knee/ (51355)

19 knee?.af. (114312)

20 18 or 19 (115529)

21 17 and 20 (2085)
