# Supplementary material for: Systematic review of the evidence on orthotic devices for the management of knee instability related to neuromuscular and central nervous system disorders
Source: BMJ Open. 2017 Sep 5;7(9):e015927. doi: 10.1136/bmjopen-2017-015927 (PMC5588970; doi:10.1136/bmjopen-2017-015927)
Supplement: Supplementary file 2 [file bmjopen-2017-015927supp002.pdf]

SUPPLEMENTARY FILE 2 QUALITY ASSESSMENT

Randomised controlled trials

Study	Selection bias Random sequence generation	Selection bias Allocation concealment	Performance bias Blinding of participants and personnel	Detection bias Blinding of healthcare professional assessed outcomes	Attrition bias	Selective outcome reporting	Other
Yang [23]	Unclear risk	Unclear risk	High risk<br><br>Not possible due to nature of intervention	High risk of bias<br>Treating clinician assessed outcome which is likely to be influenced by lack of blinding	Low risk of bias	Unclear	
Harvey[24]	Unclear risk	Unclear risk	High risk<br><br>Not possible due to nature of intervention	High risk of bias<br>Treating clinicians appeared to be involved in gathering data on outcomes likely to be influenced by lack of blinding	High risk of bias for ambulatory outcomes	Unclear	Only a small number of patients wore their second device suggesting a cross-over design was not appropriate.

Non-randomised controlled studies

Study	Selection criteria adequately reported?	Representative sample?	Participation rate ≥80%?	Performance bias?	Independent outcome assessment?	Follow-up ≥80%?	Selection bias?							
							1	2	3	4	5	6	7	8
Morinaka[22]	N	U	N	U	N	NA[a]	Y	Y	U	U	U	U	N[b]	U
Tang[32]	Y	Y	Y	U	N	U	U	U	U	U	U	U	U	U
Whittle[33]	N	U	U	U	N	Y	U	U	U	U	U	U	U	U

1 gender; 2 age; 3 cause of muscle weakness; 4 presence of sensory disturbance; 5 purpose of orthosiss (proximal/distal muscle weakness); 6 previous use of orthosis; 7 acclimatisation time; 8 type of orthosis used

a Appears to be retrospective

b The average time post-stroke was 20 months for the KAFO group and 40 months for the AFO group suggesting likely differences in functioning and time using an orthotic device

Case series

Study	Selection criteria adequately reported?	Representative sample?	Participation rate ≥80%?	Prospective?	Independent outcome assessment?	Follow-up ≥80%?	Prognostic variables reported?	Co-interventions?	Measure of variability?	Other important limitations
Bernhardt[19]	N	U	U	Y	N	N	Y	N	P	Reporting of results
Bocker[10]	Y	Y	U	Y	N	N	N	Y	Y	Reporting of results
Boudarham[20]	Y	U	U	Y	N	U	Y	N	Y	
Brehm[12]	Y	U	U	Y	N	Y	Y	N	P	Reporting of results
Davis[13]	Y	U	U	Y	N	Y	Y	Y	Y	Generalisability of assessing two different modes of using orthosis in clinic
Hachisuka[14]	U	Y	U	Y	N	N	Y	Y	P	Reporting of results
Heim[16]	N	U	U	Y	N	Y	N	N	N	Reporting of results
Jaspers[27]	N	U	N	N	Y	N	Y	Y	NA	
Kakurai[21]	N	U	U	Y	N	Y	Y	N	Y	Ability to actively control knee a confounder for KAFO and AFO comparisons
Middleton[28]	Y	U	U	U	N	Y[a]	Y	Y	Y	Only patients who had successfully completed gait training and

										continued to used the orthosis were administered a questionnaire
Peethambaran[17]	Y	U	U	Y	N	Y	Y	N	Y	Generalisability due to small sample
Scivoletto	U	U	U	Y	N	Y[a]	Y	N	Y	
Steinfeldt[18]	N	U	U	N	N	Y	N	N	N	
Summers[30]	Y	U	U	N	N	NA[b]	N	Y	NA	Lack of information on interview questionnaire
Sun[31]	N	U	U	U	N	N	N	U	N	
Wu[34]	Y	U	U	U	N	Y	Y	Y	Y	Generalisability due to small sample

N, no; NA, not applicable; P, partial; Y, yes; U, unclear

a For outcome/s included in review; b Retrospective study
