# Supplementary material for: Systematic review of the evidence on orthotic devices for the management of knee instability related to neuromuscular and central nervous system disorders
Source: BMJ Open. 2017 Sep 5;7(9):e015927. doi: 10.1136/bmjopen-2017-015927 (PMC5588970; doi:10.1136/bmjopen-2017-015927)
Supplement: Author's manuscript [file bmjopen-2017-015927.draft_revisions.pdf]

BMJ Open is committed to open peer review. As part of this commitment we make the peer review history of every article we publish publicly available.

When an article is published we post the peer reviewers' comments and the authors' responses online. We also post the versions of the paper that were used during peer review. These are the versions that the peer review comments apply to.

The versions of the paper that follow are the versions that were submitted during the peer review process. They are not the versions of record or the final published versions. They should not be cited or distributed as the published version of this manuscript.

BMJ Open is an open access journal and the full, final, typeset and author-corrected version of record of the manuscript is available on our site with no access controls, subscription charges or pay-per-view fees (http://bmjopen.bmj.com).

If you have any questions on BMJ Open's open peer review process please email editorial.bmjopen@bmj.com

BMJ Open

A SYSTEMATIC REVIEW OF THE EVIDENCE ON ORTHOTIC DEVICES FOR THE MANAGEMENT OF KNEE INSTABILITY RELATED TO NEUROMUSCULAR AND CENTRAL NERVOUS SYSTEM DISORDERS

Journal:	*BMJ Open*
Manuscript ID	bmjopen-2017-015927
Article Type:	Research
Date Submitted by the Author:	10-Jan-2017
Complete List of Authors:	McDaid, Catriona; University of York, Department of Health Sciences<br>Fayter, Debra; University of York, Centre for Reviews and Dissemination; Kleijnen Systematic Reviews Ltd<br>Booth, Alison; York University, Department of Health Sciences<br>O'Connor, Joanne; Newcastle University, Institute of Health and Society<br>Rodriguez-Lopez, Rocio; University of Leeds, Academic Unit of Health Economics<br>Mccaughan, Dorothy; University of York, Department of Health Sciences<br>Bowers, RJ; University of Strathclyde<br>Iglesias, C; University of York, Department of Health Sciences<br>Lalor, Simon; Queen Mary's Hospital<br>O'Connor, Rory J.; University of Leeds<br>Phillips, Margaret Philip; Royal Derby Hospital<br>Ramdharry, Gita; Kingston University; St George's University of London
**Primary Subject Heading**:	Rehabilitation medicine
Secondary Subject Heading:	Evidence based practice
Keywords:	systematic review, orthotic device, knee instability, central nervous system disorder, Neuromuscular disease < NEUROLOGY

SCHOLARONE™
Manuscripts

A SYSTEMATIC REVIEW OF THE EVIDENCE ON ORTHOTIC DEVICES FOR THE MANAGEMENT OF KNEE INSTABILITY RELATED TO NEUROMUSCULAR AND CENTRAL NERVOUS SYSTEM DISORDERS

Catriona McDaid[1], Debra Fayter[2], Alison Booth[1], Joanne O'Connor[3], Rocio Rodriguez-Lopez[4], Dorothy McCaughan[1], Roy Bowers[5], Cynthia P Iglesias[1], Simon Lalor[6], Rory J O'Connor[7], Margaret Phillips[8], Gita Ramdharry[9]

[1]Department of Health Sciences, University of York, York, UK

[2]Centre for Reviews & Dissemination, University of York, York, UK

[3]Institute of Health and Society, Newcastle University, Newcastle Upon Tyne, UK

[4]Academic Unit of Health Economics, University of Leeds, Leeds, UK

[5]Biomedical Engineering, University of Strathclyde, Glasgow, UK

[6]Queen Mary's Hospital, St George's University Hospitals NHS Foundation Trust, London, UK

[7]Leeds Institute of Rheumatic and Musculoskeletal Medicine, University of Leeds, Leeds, UK

[8]Royal Derby Hospital, Derby, UK

[9]Kingston University and St George's University of London, London, UK

Correspondence to:

Dr Catriona McDaid, York Trials Unit, Dept. of Health Sciences, ARRC Building, University of York, York, YO10 5DD; catriona.mcdaid@york.ac.uk

Word Count: 3824

Keywords: systematic review, orthotic devices, knee instability, central nervous system disorder, neuromuscular disease

For peer review only

ABSTRACT

Objectives: To assess the effectiveness of orthotic devices for the management of instability of the knee in adults with a neuromuscular or central nervous system disorder

Design: A systematic review of primary studies

Setting: Community

Participants: Adults with a neuromuscular or central nervous system disorder and impaired walking ability due to instability of the knee

Interventions: Orthoses with the clinical aim of controlling knee instability for example, knee-ankle-foot orthoses (KAFO), ankle-foot orthoses (AFO) and knee orthoses (KO) or mixed design with no restrictions in design or material.

Primary and secondary outcome measures: condition specific or generic patient-reported outcome measures assessing function, disability, independence, activities of daily living, quality of life or psychosocial outcomes; pain; walking ability; functional assessments; biomechanical analysis; adverse effects; usage; patient satisfaction and the acceptability of a device; and resource utilisation data.

Results: Twenty-one studies including 478 patients were included. Orthotic devices were evaluated in patients who were post-polio, post-stroke, with inclusion body myositis and with spinal cord injury. The review included two randomised controlled trials, three non-randomised controlled studies and 16 case series. Most were small, single centre studies with only six of 21 following patients for one year or longer. They met between one and five of nine quality criteria and reported methods and results poorly. They mainly assessed outcomes related to gait analysis and energy consumption with limited use of standardised, validated patient-reported outcome measures. There was an absence of evidence on outcomes of direct importance to patients such as reduction in pain and falls.

Conclusions: There is a need for high quality research, particularly RCTs, of orthotic devices for knee instability related to neuromuscular and central nervous system conditions. This research should address outcomes important to patients. There may also be value in developing a national registry.

Registration number systematic review: PROSPERO (CRD42014010180).

For peer review only - http://bmjopen.bmj.com/site/about/guidelines.xhtml

ARTICLE SUMMARY

Strengths and limitations of this study

- The first systematic review addressing this question to systematically consider study quality.
- An extensive range of sources were searched to identify studies.
- It was difficult to be certain that knee instability was the main problem being treated in some of the studies.
- Due to poor reporting of the primary studies, it was not possible to extract outcome data in the standardised way planned in the protocol.

For peer review only

For peer review only - http://bmjopen.bmj.com/site/about/guidelines.xhtml

INTRODUCTION

Instability can occur in any of the three anatomical planes of the knee: sagittal, coronal or transverse planes and there are several mechanisms that may lead to knee instability in neuromuscular disorders (NMD) and central nervous system (CNS) conditions. These include: weakness or over-activity of any of the muscles that have a direct effect on the knee (knee extensors, knee flexors, gastrocnemius); and muscle weakness or over-activity remote from the muscles directly affecting the knee due to secondary effects on posture (e.g. alterations to the anterior progression of the ground reaction force under the foot, or plantarflexor weakness leading to uncontrolled dorsiflexion). In the case of CNS conditions, spasticity in the muscles around the knee can also cause knee instability (e.g. spasticity in the gastrocnemius causes excessive plantarflexion in stance which shifts the ground reaction force anterior to the knee causing hyperextension).[1] Knee instability can lead to pain, falls and a range of mobility issues for the individual.

Knee instability due to muscle weakness or ligamentous laxity is often treated using orthoses with the functional goals of improving walking and to protect, stabilise and improve function.[2] Knee orthoses (KO) are often prescribed or in some cases a type of ankle-foot orthosis (AFO) known as a Ground Reaction AFO (GRAFO) may be provided. A GRAFO provides direct control of the ankle and foot, and indirect control of the knee and hip may be provided through optimising and normalising the alignment of the Ground Reaction Force in relation to the knee joint throughout stance phase. A knee-ankle-foot orthosis (KAFO) is usually prescribed when bracing with an AFO or KO is insufficient to adequately control knee instability and usually when control in more than one plane is required.[2] Modern KAFOs tend to combine plastic and metal components: commonly polypropylene for calf and thigh shells and shoe inserts, aluminium, magnesium, titanium or steel for uprights and steel for joints.[3] Variations exist in the orthotic knee joint design, locking and unlocking mechanism, type of knee pads and plane of control.[3] A locked knee KAFO requires an altered gait to allow the individual's foot to clear the ground in the swing phase of walking. Polycentric knee joints can be locked or unlocked and permit a more anatomical or natural knee motion, though have more two-joint axes and may require more maintenance and are therefore more expensive.[3] Stance control knee joints have either a mechanical or microprocessor controlled knee joint which allows the knee to flex during the swing phase of walking, but lock during the stance phase of walking, when the knee is extended, allowing a more normal walking pattern. Other, more extensive options include hip-knee-ankle-foot orthoses (HKAFO) originally designed for patients with higher level spinal cord dysfunction who might otherwise have been

For peer review only - http://bmjopen.bmj.com/site/about/guidelines.xhtml

unable to walk.[4] These include hip guidance orthoses (HGOs) and reciprocating gait orthoses (RGOs) which have different locking mechanisms.

We undertook a systematic review with the aim of assessing the evidence base for the effectiveness of orthotic devices for management of instability of the knee in adults who have NMD or a CNS disorder. This was part of a larger mixed-methods project undertaken to inform the development of a future substantive research question on the clinical and cost-effectiveness of different types of orthotic management of the knee in people with NMD or CNS disorders.[1]

METHODS

Search methods for identification of studies

We searched the following databases from inception to November 2014: MEDLINE via Ovid, MEDLINE In-Process via Ovid, Cumulative Index to Nursing & Allied Health (CINAHL) via EBSCO, EMBASE via Ovid, PASCAL via Ebsco, Scopus, Science Citation Index (ISI Web of Knowledge), BIOSIS Previews, PEDro, Recal Legacy, Cochrane Database of Systematic Reviews (CDSR), Database of Abstracts of Reviews of Effects (DARE), Health Technology Assessment (HTA) database and Cochrane Central Register of Controlled Trials (CENTRAL) in The Cochrane Library , Conference Proceedings Citation Index- Science (CPCI-S) (ISI Web of Knowledge), Health Management Information Consortium (HMIC) via Ovid, ClinicalTrials.gov, International Clinical Trials Registry Platform (WHO ICRTP) National Technical Information Service (NTIS) and selected websites. There were no language or publication status restrictions. See online supplementary appendix 1 for the MEDLINE (Ovid) search strategy which was adapted for the other databases.

The reference lists of all included studies, any related systematic reviews and key background papers were checked to identify any further, relevant studies.

Eligibility criteria

Population: Adults (16 years or older) with a neuromuscular or central nervous system disorder and impaired walking ability due to instability of the knee were eligible for inclusion.

Intervention: Orthoses with the clinical aim of controlling knee instability for example, knee-ankle-foot orthoses (KAFO), ankle-foot orthoses (AFO) and knee orthoses (KO) or of mixed design with no restrictions in design, material, custom or pre-fabricated; type of knee joint or stance-control design

For peer review only - http://bmjopen.bmj.com/site/about/guidelines.xhtml

For peer review only

(KAFO), or whether there was an electronic component. Studies evaluating the use of functional electrical stimulation were excluded.

Studies were eligible provided the orthosis had been used in a real-life setting (i.e. not solely within a laboratory / experimental setting). Outcomes could be assessed in a laboratory or clinic setting provided participants had used the device in the community.

Comparator: Studies using any of the above orthoses as a comparator; including studies comparing different designs of the same orthosis; or no intervention.

Study design: Randomised controlled trials (RCTs) and other study designs with and without a comparator group such as non-randomised controlled studies, before and after studies and case series were eligible for the review.

The following outcomes were of interest: Condition specific or generic patient-reported outcomes measures assessing function, disability, independence, activities of daily living, quality of life or psychosocial outcomes; pain; walking ability; functional assessments; biomechanical analysis; adverse effects; usage; patient satisfaction and the acceptability of a device; and resource utilisation data.

Two researchers independently screened titles and abstracts and full papers to assess eligibility. Disagreements were resolved through discussion and consultation with another member of the project team if necessary. Authors were contacted if eligibility was uncertain from the information provided in the publication. There were no language restrictions.

Data extraction

Data were extracted by one researcher and checked by a second with discrepancies resolved by discussion. Studies in languages other than English were extracted by a native speaker who was also a researcher and were checked by a second researcher for consistency only. Data were extracted using a piloted data extraction form. Multiple publications of the same study (linked papers) were extracted and reported as a single study. Between-group differences were extracted from studies with a comparator. We had planned to extract data to allow calculation of between group differences and confidence intervals. However due to the generally poor reporting of data, it was not possible to consistently do this across studies. Where data were available, these were extracted; where the appropriate data were not reported, the description of the results provided in the paper was extracted and the lack of summary data noted.

Study quality

For peer review only

RCTs were assessed using the Cochrane risk of bias criteria.[5] Non-randomised studies with a control group were assessed for external validity, performance bias, detection bias and selection bias/control of confounding based on eight criteria (gender, age, cause of muscle weakness, presence of sensory disturbance, whether the orthosis was used for proximal or distal muscle weakness, previous use of an orthosis, acclimatisation time and type of orthosis used). Case series were assessed using criteria adapted from the assessment of controlled studies and criteria used in a previous systematic review.[6] Assessment of risk of bias was undertaken independently by two researchers (except for non-English language studies). Discrepancies were resolved by discussion.

The protocol was registered with PROSPERO in advance of undertaking the review (registration number CRD42014010180). Ethical approval was not required.

RESULTS

Overview of the evidence

A total of 4516 references were identified from the searches and 21 studies of 478 patients (reported in 25 publications) were included (Figure 1). A full list of papers and reasons for exclusion is available from the authors. A substantial proportion were excluded (n=76) because the orthosis was evaluated in a laboratory or clinical setting without the participant using the device in the community. Three potentially relevant ongoing studies were identified: a before and after study;[7] a case series,[8] and an RCT.[9]

Table 1 provides a summary of study characteristics grouped by the four conditions covered by the included studies: post-polio, inclusion body myositis, post stroke, and spinal cord injury. Two RCTs, three non-randomised studies with a control group, and 16 case series were included. Sample sizes ranged from five to 67 participants and just fewer than half the studies had over 20 participants. The follow-up time was generally short, only 6 studies followed patients for one year or longer.

For peer review only - http://bmjopen.bmj.com/site/about/guidelines.xhtml

Table 1: Study characteristics by condition

Main publication (*associated papers*) Country	Study design N in study (n in analyses)	Population % male Mean age (SD)	Intervention (I) Comparator (C)	Co-interventions	Length of follow-up
			**POST-POLIO SYNDROME**		
Bocker, 2013[10] (*Bocker, 2011[11]*) Germany	Case series N=10 (6)	30% 64.5	I: Carbon fibre KAFO C: No comparator	Gait training, pain therapy and exercises (twice per week for 3 months)	3 months
Brehm, 2007[12] Netherlands	Case series N=23 (20)	61% 55 (9.2)	I: Carbon fibre KAFO (locked knee-joint) C: Leather /metal or plastic /metal KAFO used previously by same participants	Walking aids were used by some participants	26 weeks
Davis, 2010[13] Australia	Case series N=10 (10)	40% 61.9 (7.7)	I: Carbon fibre SCKAFO in stance control mode C: KAFO in locked knee mode used by same participants	Walking aids	Mean duration of use at time of evaluation 6.2 (SD 5.2) months
Hachisuka, 2006[14] Japan (*Hachisuka, 2007 [15]*)	Case series N=11 (8 to 11[a])	18% 53.9 (9.8)	I: Carbon fibre KAFO C: Traditional non-carbon KAFO used by same participants	Walking aids	Not reported
Heim, 1997[16] Israel	Case series N=30 (27)	33% 44	I: Carbon fibre KAFO C: No comparator	Not reported	30 months
Peethambaran, 2000[17] USA	Case series N=5 (5)	40% 61.4 (12.4)	I: Carbon titanium KAFO (anterior approach design) C: Plastic KAFO (posterior approach design) used previously by the same participants	Not reported	6 weeks

For peer review only - http://bmjopen.bmj.com/site/about/guidelines.xhtml

Main publication (*associated papers*) Country	Study design N in study (n in analyses)	Population % male Mean age (SD)	Intervention (I) Comparator (C)	Co-interventions	Length of follow-up
Steinfeldt, 2003[18] Germany	Case series N=55 (55)	44% 58	I: Carbon fibre KAFO C: No comparator	Not reported	>3 months
			**INCLUSION BODY MYOSITIS**		
Bernhardt, 2011[19] USA	Case series N=9 (6)	78% 61 (9)	I: SCKAFO C: No comparator	Not reported	6 months
			**POST-STROKE**		
Boudharam, 2013[20] France	Case series N=11 (unclear)	64% 51 (15)	I: Carbon fibre KAFO C: No comparator	Not reported	Device prescribed within past 6 months
Kakurai, 1996[21] Japan	Case series N=28 (28)	50% 54.5	I: Plastic convertible KAFO (to AFO) C: Participants who changed to AFO compared with those remaining on KAFO	Not reported	Not reported
Morinaka, 1982[22] Japan	Cohort study N=25 (25)	64% 56	I: Plastic KAFO C: 50 participants fitted with AFOs and a group of 30 healthy adult males	Not reported	Mean 14.6 months (range 1 to 35)
Yang , 2005[23] China	RCT N=67 (67)	84% 58	I: KAFO or AFO C: 'Conventional rehabilitation'	Not reported	Not reported

For peer review only - http://bmjopen.bmj.com/site/about/guidelines.xhtml

**Main publication (*associated papers*) Country**	**Study design N in study (n in analyses)**	**Population % male Mean age (SD)**	**Intervention (I) Comparator (C)**	**Co-interventions**	**Length of follow-up**
			**SPINAL CORD INJURY**		
Harvey, 1997[24] Australia (*Harvey, 1997;*[25] *Harvey, 1998* [26])	RCT (cross-over) N=10 (5-10[b])	90% 37 (8.4)	I: HKAFO (Walkabout Orthosis) C: HKAFO (IRGO)	Gait training (30 to 54 hours per orthosis) Crutches	28 weeks
Jaspers, 1997[27] Belgium	Case series N=14 (14)	86% 33.6	I: HKAFO (ARGO) C: No comparator	Walker or Crutches	1 year
Middleton, 1997[28] Australia	Case series N=25 (21)	76% 35 (13)	I: HKAFO (Walkabout Orthosis) C: No comparator	Parallel bars, forearm crutches or frames	≥ 18 months
Scivoletto, 2000[29] Italy	Case series N=24 (24[c])	79% 33.6 (3.2)	I: HKAFO (RGO) C: No comparator (internal comparison of non-users versus users)	Not reported	1 year
Summers, 1988[30] UK	Case series N=20 (20)	100% 28	I: HKAFO (HGO ParaWalker) C: No comparator	Crutches used as decided by patient	Mean 20 months
Sun, 2007[31] China	Case series N=20 (15)	67% 33.7	I: HKAFO (RGO) C: No comparator	Not reported	Not reported

For peer review only - http://bmjopen.bmj.com/site/about/guidelines.xhtml

Main publication (*associated papers*) Country	Study design N in study (n in analyses)	Population % male Mean age (SD)	Intervention (I) Comparator (C)	Co-interventions	Length of follow-up
Tang, 2009[32] China	Controlled study N=58 (unclear)	83% 32.4	I: AGO, RGO, KAFO C: Rehabilitation training	Rehabilitation training	4 months[d]
Whittle, 1991[33] UK	Controlled study (cross-over) N=22 (Unclear[e])	82% 34	I: HKAFO (HGO ParaWalker) C: HKAFO (RGO)	Rollator or crutches	4 months
Wu, 2003[34] China	Case series N=6 (6)	67% 27.6	I: HKAFO (Walkabout Orthosis) C: No comparator group	Gait training including balance plus walking exercises	Unclear

SD, Standard deviation

[a] Eight completed assessment of non-carbon fibre KAFO and walking without an orthosis and 11 completed assessment of carbon-fibre KAFO

[b] Appears to be 22 for analysis of final choice of orthosis, although one left the trial without trying either and three participants tried only one. It was unclear how many participants were included in other analyses

[c] Appears to be 10 for all analyses except for speed of walking on flat surface (n=8) and speed of walking on ramp (n=5)

[d] Eight weeks after fitting of device

[e] A total of 24 for the single outcome eligible for the review, although unclear for other analyses in the study

For peer review only - http://bmjopen.bmj.com/site/about/guidelines.xhtml

Study Quality

Both RCTs were assessed as having an unclear or high risk of bias for the majority of items on the Cochrane risk of bias tool (Supplementary file 2). Overall the three non-randomised controlled studies were at risk of selection bias (Supplementary file 2). Ten of the 16 studies without a control group were prospective, three were retrospective and this aspect of the design was unclear for three. Overall the 16 studies met between one and five of the nine quality criteria, only eight adequately described their inclusion criteria and all were considered at risk of selection bias (Supplementary file 2). Poor reporting of study methods and results was a problem across all the study designs. Studies often made statements in the results section which were not backed up with numerical data. Where data were available, these were extracted; where the appropriate data were not reported, the description of the results provided in the paper was extracted and the lack of summary data noted in the data extraction tables which are available in the HTA report.[1]

Outcomes

The most systematically assessed outcomes in the included studies were gait quality and energy consumption, assessed during clinic/laboratory visits (Table 2). While several studies (Table 2) reported patient satisfaction with the device and functionality (e.g. how it impacted sitting in their wheelchair; the main ways in which they used the device), the results were predominantly reported in an anecdotal fashion and it was not possible to assess how robustly the information had been collected. Despite our requirement that participants in studies had used their orthoses outside the clinic only one study used a validated measure (Barthel Index) of patients' ability to manage every day activities of daily living outside the clinic setting;[21] and only two assessed quality of life using a validated measure (Table 2). Generally, adverse effects such as skin damage or falls were not systematically reported. It cannot be inferred that there were few adverse events as authors did not specifically mention that no adverse events were identified.

For peer review only

Table 2: Outcomes assessed

	Patient-reported outcomes					*Objective assessments*			*Resource utilisation*	
**Study**	**Satisfaction with device**	**Functionality of device**	**Usage of device**	**Quality of life**	**Adverse effects**	**Walking ability**	**Energy consumption**	**Muscle activity**	**Device malfunction**	**Cost**
**POST-POLIO**										
Bocker[10]				✓				✓		
Brehm[12]	✓					✓	✓		✓	
Davis[13]						✓	✓			
Hachisuka[14]	✓					✓	✓		✓	✓
Heim[16]	✓		✓		✓				✓	
Peethambaran[17]	✓	✓			✓					
Steinfeldt[18]	✓	✓							✓	
**INCLUSION BODY MYOSITIS**										
Bernhardt[19]	✓	✓	✓			✓				
**POST STROKE**										
Boudharam[20]						✓				
Kakurai[21]						✓		✓		
Morinaka[22]			✓			✓				
Yang[23]								✓		
**SPINAL CORD INJURY**										
Harvey[24]	✓	✓	✓			✓	✓	✓		
Jaspers[27]	✓	✓	✓		✓				✓	
Middleton[28]	✓	✓	✓						✓	
Scivoletto[29]		✓	✓							
Summers[30]	✓	✓	✓		✓				✓	
Sun[31]		✓			✓	✓				

For peer review only - http://bmjopen.bmj.com/site/about/guidelines.xhtml

Tang[32]		✓		✓						
Whittle[33]	✓	✓		✓		✓			✓	✓
Wu[34]		✓				✓		✓		

For peer review only

Post-polio patients

Seven case series (n=143 patients) investigated types of carbon fibre KAFO (Table 1). Three compared a new device to the one used previously by participants;[12,14,17] one compared using a device in stance control mode and locked mode (with the aim of replicating a traditional KAFO design);[13] two before and after use of the orthosis;[10,18] and post-intervention only in one study.[16]

Outcomes were sparsely reported. Five of the seven studies reported measures of patient satisfaction, although not in sufficient detail to assess the robustness of the evaluation.[12,14,16-18] Three studies made a formal assessment of walking ability,[12-14] and four assessed either energy consumption or particular muscle activity.[10,12-14] Resource utilisation data was limited to assessment of device malfunction in four studies[12,14,16,17] and cost in one study.[14] Five studies failed to report adverse effects data or to mention that no adverse effects were identified.[10,12-14,18]

Inclusion body myositis

One case series study (n=9 patients) evaluated a stance control KAFO. [19] Gait was assessed in the clinic with and without use of the device following six months of use. A questionnaire was designed by the investigators to elicit patient outcomes but the results were not reported in full. No data were reported on resource utilisation or adverse effects.

Post-stroke patients

Four studies (n=131 patients); one RCT,[23] a cohort study,[22] and two case series[20,21]evaluated KAFOs and/or AFOs used for knee instability. One assessed a single outcome, gait with and without use of a carbon fibre KAFO. [20] Two studies compared a thermoplastic KAFO to an AFO for knee instability: one compared patients who had changed to an AFO with those who continued to use a KAFO,[21] effectively the comparison was between those who had recovered sufficient control of knee activity to switch to an AFO compared to those who had not; the second compared KAFO to AFO and to normal adult gait.[22] The RCT compared use of an AFO or KAFO to what was described as conventional rehabilitation (not reported in detail).[23] The only patient reported outcome assessed was usage.[22] Three studies made a formal assessment of walking ability,[20-22] and two assessed other functional abilities.[21,23] None reported on resource utilisation data or adverse effects.

Patients with spinal cord injury

For peer review only - http://bmjopen.bmj.com/site/about/guidelines.xhtml

Nine studies (n=194 patients); one RCT[24] two controlled trials,[32,33] and six case series,[27-31,34] evaluated HKAFOs. The RCT used a cross-over design to compare Walkabout® Orthosis (WO) (Polymedic, Queensland Australia) to an isocentric® RGO (IRGO) (Center for Orthotics Design, Campbell, CA). There was a two month washout period of no orthoses use; the data were analysed as though from a parallel trial.[24] There were two further studies of WO with no comparator.[28,34] Two investigated a HGO, one with no comparator,[30] and one compared the HGO to a custom made RGO worn by the same patients in a crossover study.[33] The remaining four studies investigated types of RGO, two with no comparator,[27,31] one in comparison with RGO non-users,[29] and one compared three different types of orthoses (plus rehabilitation training) to rehabilitation training alone.[32] Although each of the studies reported at least one patient-reported outcome, only one study reported using a validated scale (Barthel Index and Functional Independence Measure); and due to lack of clarity in the analysis and reporting it is unclear whether there were any between group differences at follow-up in this study. [32] There were fewer objective assessments across the studies than for the other conditions.[24,31,33,34] Resource utilisation data was limited to assessment of device malfunction [27,28,30,33] and cost.[33] Two thirds of studies did not address adverse effects.[24,28,29,32-34]

DISCUSSION

Principal findings

The review identified a paucity of high quality evidence assessing the effectiveness of orthotic devices for knee instability experienced by people with neuromuscular and central nervous system conditions. In addition to the very limited use of robust study designs, in particular RCTs, reporting was generally poor. For example several studies made statements about findings without presentation of supporting data. The evidence base consists of small, single centre studies with outcome assessments that did not appear to have been undertaken independently of treating clinicians. Laboratory-based studies can provide useful insights about efficacy, particularly during development of a device. However the literature is dominated by laboratory evaluations of orthoses: 76 studies were excluded because the evaluation of the orthosis did not include any use of the device by the patient in a non-clinic setting and the most systematically assessed outcomes in the included studies focused on gait analysis and energy consumption. There was limited use of standardised, validated patient reported outcome measures. In particular there was an absence of evidence on outcomes that are reported by patients to be important to them such as reduction in pain, falls or trips, improved balance and stability and participation in paid employment, outdoor

For peer review only

activities (such as gardening), family visits and social events. [1] In addition, fewer than one third of the studies followed patients for a year or more. It is unlikely that studies of less than one year duration fully capture the effects of using the devices.

Given that patients report that orthotic devices prescribed for knee instability can play a crucial role in maintaining, promoting and enhancing physical and psychological health and well-being and participation in employment, family and social community activities, [1] the evidence gaps identified by our review are significant and important. A factor that might contribute to this discrepancy in outcome measurement is current requirements for device regulation; only evidence of performance and safety is required for medical devices associated with lower levels of risk to patients such as orthotics for knee instability. This may result in a lack of incentives to conduct primary research on efficacy and/or effectiveness.[35]

Strengths and weaknesses of the study

We undertook systematic searches across an extensive range of sources for published, unpublished and ongoing studies. There were no language restrictions and we included three studies published in Chinese[23,31,32] and one in German.[18] We assessed the risk of bias in the included studies and used standard methods to reduce error and bias at key stages of the review process. Several studies provided a descriptive report of some outcomes with no numerical data. Due to the paucity of evidence we extracted these reports in order to provide as clear a picture as possible of what information is currently available. Arguably this overestimates the amount of evidence that is available.

During study selection it was often difficult to definitively determine whether the participants had knee instability. This was partly due to poor reporting and partly because knee instability was sometimes part of a more complex problem with stability and mobility and is not an explicit and well defined clinical diagnosis. As a result studies may have been included where it is arguable whether knee instability was the main problem and studies rejected that arguably do include people with knee instability. However, we would not expect that this would in any way change the overall conclusions of the review about the lack of high quality evidence or allow conclusions to be made about the effectiveness of specific devices.

An evidence base of small single-centre studies and inadequate study design is similar to that identified in other reviews of orthotic devices for different populations.[36] [37] [38] Also a systematic review of questionnaires used to assess patient satisfaction with orthoses for any limb

For peer review only - http://bmjopen.bmj.com/site/about/guidelines.xhtml

found that 63% of the 106 included papers used questionnaires developed for the specific study rather than validated measures, supporting our findings on this aspect of the evidence.[39]

Unanswered questions and future research

There is a large gap in the evidence on the effectiveness of KAFOs, AFOs and other orthotic devices for managing knee instability related to NMD and CNS conditions. Robust research is required addressing outcomes that are important to patients. RCTs are the most robust way of assessing effectiveness and a pragmatic trial that recognises that provision of an orthotic device is a complex intervention would be appropriate.

There are a number of challenges for researchers and clinicians to consider when designing future studies, including: defining the target population and knee instability, the personalisation of treatment including customisation of devices, the relative rarity of the problem within individual conditions and whether a trial including patients with knee instability with a range of NMD or CNS conditions would be generalisable. It may also be worth considering a national registry to systematically collect data on the ambulatory problem, devices provided, key elements of management of the instability, factors that inform / determine the process of matching patients to orthotic devices, collection of a core set of standardised and validated patient-reported outcome measures, data on use of the device and resource use. While registries do have limitations this would be a major step change from the current evidence base in terms of increased rigour and generalisability and would create a population database and an infrastructure from which future RCTs could be undertaken. The evidence base in this field could also be improved through systematic development of a core set of outcome measures (http://www.comet-initiative.org/). Future research regardless of study design should follow reporting standards (http://www.equator-network.org/).

CONCLUSIONS

There is a need for high quality research, in particular RCTs, on the effectiveness of KAFOs, AFOs and other orthotic devices for managing knee instability related to NMD and CNS conditions. This research should address outcomes that are important to patients. There may also be value in developing a national registry.

For peer review only - http://bmjopen.bmj.com/site/about/guidelines.xhtml

COMPETING INTERESTS

During this study Simon Lalor was an employee of Opcare, a company that provides orthotic and prosthetic services to the UK NHS. This company does not manufacture orthotic devices, although a sister company ORTHO C FAB does. Cynthia Iglesias is a member of the National Institute for Health and Care Excellence Medical Technologies Assessment Committee and member of the European Clinical Research Infrastructure Network.

AUTHOR'S CONTRIBUTIONS

CMcD was responsible for writing the protocol and had overall responsibility for co-ordinating and leading the project, provided advice and input to all elements of the project and contributed to report writing. AB, DF, JO'C undertook study selection, quality assessment, report writing and contributed to the protocol. RRL provided information specialist support, designed and undertook literature searches and wrote the related sections in the report

RB, CPI, SL, MP, GR, DMcC, and RJO'C were members of the Advisory Group, contributed to the systematic review protocol and/or provided clinical and/or methodological advice throughout the review and commented on drafts of the systematic review report.

CMcD drafted this manuscript and all authors reviewed, edited and approved.

FUNDING STATEMENT

This project was funded by the National Institute for Health Research (NIHR) HTA Programme (project number 13/30/02) and has been published in full in *Health Technol Assess* 2016;20(55). Further information available at https://www.journalslibrary.nihr.ac.uk/programmes/hta/133002/#/

This report presents independent research commissioned by the NIHR. The views and opinions expressed by authors in this publication are those of the authors and do not necessarily reflect those of the NHS, the NIHR, MRC, CCF, NETSCC, the HTA programme or the Department of Health.

DATA SHARING STATEMENT

Most of the data are available in the main body and appendices of the HTA Monograph https://www.journalslibrary.nihr.ac.uk/hta/hta20550/#/abstract
Any further data can be obtained from the corresponding author.

For peer review only - http://bmjopen.bmj.com/site/about/guidelines.xhtml

REFERENCES

1. O'Connor J, McCaughan D, McDaid C, et al. Orthotic management of instability of the knee related to neuromuscular and central nervous system disorders: systematic review, qualitative study, survey and costing analysis. Health Technol Assess 2016;**20**(55)
2. Hebert JS. Ambulatory KAFOs: a physiatry perspective. Journal of Prosthetics & Orthotics 2006;**74**(30 ref)
3. Edelstein J, Bruckner J. *Orthotics: A Comprhensive Clinical Approach*. New Jersey, USA: Slack Incorporated, 2002.
4. Campbell JH. Linked hip-knee-ankle-foot orthoses designed for reciprocal gait. Journal of Prosthetics and Orthotics 2006;**18**(7 PROCEEDINGS):P204-P08
5. Higgins J, Altman D, Sterne J. Chapter 8:Assessing risk of bias in included studies. In: Higgins J, Grees S, eds. Cochrane Handbook for Systematic Reviews of Interventions Version 5.1.0 (updated March 2011): The Cochrane Collaboration, 2011.
6. Llewellyn A, Norman G, Harden M, et al. Interventions for adult Eustachian tube dysfunction: a systematic review. Health Technol Assess 2014;**18**(46)
7. ClinicalTrials.gov. The Use of Brace to Retrain Hemiparetic Gait. URL: http://ClinicalTrials.gov/show/NCT02082938 (accessed 23 May 2016).
8. Kannenberg A, Probsting E. An orthotronic mobility system improves perceived walking capabilities in traditional leg orthosis users. PM and R 2014;**1)**:S229-S30
9. Frechtel A, Portnoy S, Raveh E, et al. Prevention of knee hyperextension in stroke patients using a knee orthosis: 3D computational gait analysis and dynamic EMG. Gait Posture 2013;**38**:S85. http://dx.doi.org/10.1016/j.gaitpost.2013.07.178
10. Bocker B, Hoelig C, Smolenski UC. Orthosis Management in Patients after Poliomyelitis Anterior Acuta. Physikalische Medizin Rehabilitationsmedizin Kurortmedizin 2013;**23**(1):16-21 doi: 10.1055/s-0032-1331218[published Online First: Epub Date]|.
11. Bocker B, Hölig C, Smolenski UC. Orthosis management in patients after poliomyelitis anterior acuta. J Rehabil Med 2011;**49**:40
12. Brehm MA, Beelen A, Doorenbosch CA, et al. Effect of carbon-composite knee-ankle-foot orthoses on walking efficiency and gait in former polio patients. J Rehabil Med 2007;**39**(8):651-7
13. Davis PC, Bach TM, Pereira DM. The effect of stance control orthoses on gait characteristics and energy expenditure in knee-ankle-foot orthosis users. Prosthet Orthot Int 2010;**34**(2):206-15. http://dx.doi.org/10.3109/03093641003773189
14. Hachisuka K, Makino K, Wada F, et al. Clinical application of carbon fibre reinforced plastic leg orthosis for polio survivors and its advantages and disadvantages. Prosthet Orthot Int2006;**30**(2):129-35
15. Hachisuka K, Makino K, Wada F, et al. Oxygen consumption, oxygen cost and physiological cost index in polio survivors: a comparison of walking without orthosis, with an ordinary or a carbon-fibre reinforced plastic knee-ankle-foot orthosis. J Rehabil Med 2007;**39**(8):646-50
16. Heim M, Yaacobi E, Azaria M. A pilot study to determine the efficiency of lightweight carbon fibre orthoses in the management of patients suffering from post-poliomyelitis syndrome. Clin Rehabil 1997;**11**(4):302-5
17. Peethambaran A. The relationship between performance, satisfaction, and well being for patients using anterior and posterior design knee-ankle-foot-orthosis. Journal of Prosthetics and Orthotics 2000;**12**(1):33-40
18. Steinfeldt F, Seifert W, Gunther KP. Modern carbon fibre orthoses in the management of polio patients--a critical evaluation of the functional aspects. Z Orthop Ihre Grenzgeb 2003;**141**(3):357-61
19. Bernhardt K, Oh T, Kaufman K. Stance control orthosis trial in patients with inclusion body myositis. Prosthet Orthot Int 2011;**35**(1):39-44. http://dx.doi.org/10.1177/0309364610389352

For peer review only - http://bmjopen.bmj.com/site/about/guidelines.xhtml

20. Boudarham J, Zory R, Genet F, et al. Effects of a knee-ankle-foot orthosis on gait biomechanical characteristics of paretic and non-paretic limbs in hemiplegic patients with genu recurvatum. Clin Biomech 2013;**28**(1):73-8. http://dx.doi.org/10.1016/j.clinbiomech.2012.09.007
21. Kakurai S, Akai M. Clinical experiences with a convertible thermoplastic knee-ankle-foot orthosis for post-stroke hemiplegic patients. Prosthet Orthot Int 1996;**20**(3):191-4
22. Morinaka Y, Matsuo Y, Nojima M, et al. Clinical evaluation of a knee-ankle-foot-orthosis for hemiplegic patients. Prosthet Orthot Int 1982;**6**(2):111-5
23. Yang JL, Xu YL, Wei Y, et al. Effects of lower limb orthosis therapy on the recovery of motor function in the post-stroke hemiplegic patients. [Chinese]. Chinese Journal of Clinical Rehabilitation 2005;**9**(9):6-7
24. Harvey LA, Newton-John T, Davis GM, et al. A comparison of the attitude of paraplegic individuals to the Walkabout Orthosis and the Isocentric Reciprocal Gait Orthosis. Spinal Cord 1997;**35**(9):580-84
25. Harvey LA, Smith MB, Davis GM, et al. Functional outcomes attained by T9-12 paraplegic patients with the walkabout and the isocentric reciprocal gait orthoses. Arch Phys Med Rehabil 1997;**78**(7):706-11
26. Harvey LA, Davis GM, Smith MB, et al. Energy expenditure during gait using the walkabout and isocentric reciprocal gait orthoses in persons with paraplegia. Arch Phys Med Rehabil 1998;**79**(8):945-9
27. Jaspers P, Peeraer L, Van Petegem W, et al. The use of an advanced reciprocating gait orthosis by paraplegic individuals: a follow-up study. Spinal Cord 1997;**35**(9):585-9
28. Middleton JW, Yeo JD, Blanch L, et al. Clinical evaluation of a new orthosis, the 'walkabout', for restoration of functional standing and short distance mobility in spinal paralysed individuals. Spinal Cord 1997;**35**(9):574-9
29. Scivoletto G, Petrelli A, Lucente LD, et al. One year follow up of spinal cord injury patients using a reciprocating gait orthosis: preliminary report. Spinal Cord 2000;**38**(9):555-8
30. Summers BN, McClelland MR, el Masri WS. A clinical review of the adult hip guidance orthosis (Para Walker) in traumatic paraplegics. Paraplegia 1988;**26**(1):19-26
31. Sun JL, Tang D, Ouyang YT, et al. Influence of reciprocating gait orthosis on walking function in paraplegic patients after ambulation. [Chinese]. Journal of Clinical Rehabilitative Tissue Engineering Research 2007;**11**(13):2437-40
32. Tang D, Pei G, Li K. The effects of alternative gait orthosis on activity of daily living and quality of life in patients with spinal cord injury. Chinese Journal of Rehabilitation Medicine 2009;**24**(11):985-88
33. Whittle MW, Cochrane GM, Chase AP, et al. A comparative trial of two walking systems for paralysed people. Paraplegia 1991;**29**(2):97-102
34. Wu JX, Zhou XL, Liu HL, et al. Effect of the new reciprocating gait orthosis (Walkabout orthosis) in improving paraplegic patients' independent living ability. Chinese Journal of Clinical Rehabilitation 2003;**7**(17):2469-70
35. Iglesias C. Does assessing the value for money of therapeutic medical devices require a flexible approach. Pharmacoeconomics Outcomes Research 2015;**15**(1):21-32
36. Tyson SF, Sadeghi-Demneh E, Nester CJ. A systematic review and meta-analysis of the effect of an ankle-foot orthosis on gait biomechanics after stroke. Clin Rehabil 2013;**27**(10):879-91. http://dx.doi.org/10.1177/0269215513486497
37. Fatone S. A review of the literature pertaining to KAFOs and HKAFOs for ambulation. Journal of Prosthetics and Orthotics 2006;**18**(7 Proceedings):P137-P68
38. Chisholm AE, Perry SD. Ankle-foot orthotic management in neuromuscular disorders: recommendations for future research. Disabil 2012;**7**(6):437-49. http://dx.doi.org/10.3109/17483107.2012.680940

For peer review only - http://bmjopen.bmj.com/site/about/guidelines.xhtml

39. Bettoni E, Ferriero G, Bakhsh H, et al. A systematic review of questionnaires to assess patient satisfaction with limb orthoses. Prosthet Orthot Int 2014;Published Online first:26 November 2014 doi: 10.1177/0309364614556836.

LEGEND

Figure 1 Study Selection

For peer review only

For peer review only - http://bmjopen.bmj.com/site/about/guidelines.xhtml

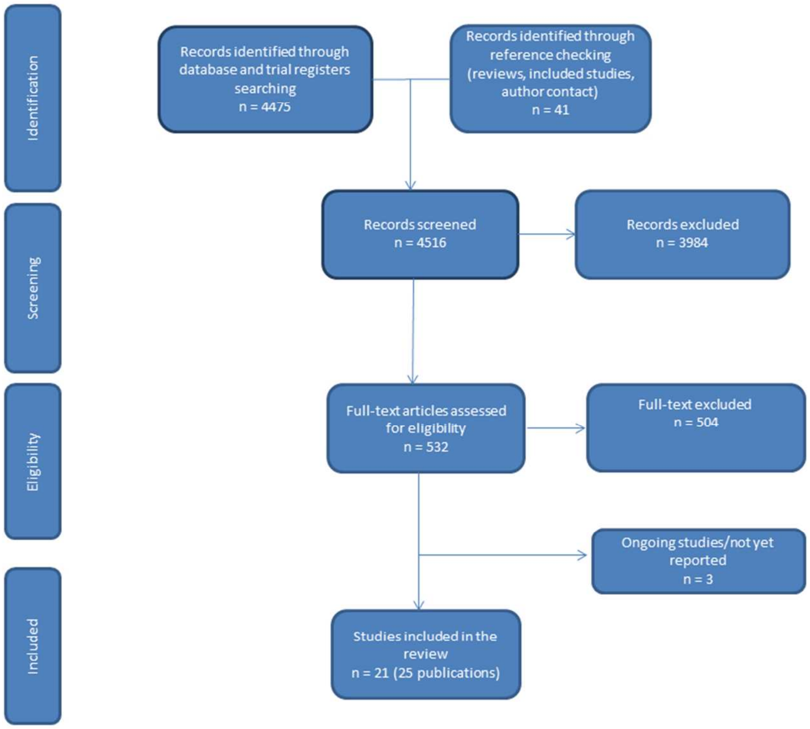

Figure1: Study selection

254x190mm (96 x 96 DPI)

SUPPLEMENTARY APPENDIX 1

Database: Ovid MEDLINE(R) In-Process & Other Non-Indexed Citations and Ovid MEDLINE(R) <1946 to Present> (searched online 22/05/14)

Search Strategy:

1 Orthotic Devices/ or Braces/ or Splints/ (16320)

2 Gait/ (17744)

3 Lower Extremity/ or Leg/ (61929)

4 Hip/ or Hip Joint/ (28943)

5 Knee/ or exp Knee Joint/ (51355)

6 Ankle/ or Ankle Joint/ (16707)

7 Foot/ or Foot Joints/ (20388)

8 1 and (2 or 3 or 4 or 5 or 6 or 7) (2732)

9 Foot Orthoses/ (145)

10 8 or 9 (2870)

11 ((gait or "lower extremity" or "lower extremities" or "lower limb" or "lower limbs" or leg? or hip? or knee? or ankle? or foot or feet) adj3 (orthos* or orthot* or brace? or bracing or support)).ti,ab. (3590)

12 (heel adj2 (pad? or raise?)).ti,ab. (365)

13 ((shoe? and (modification? or insert? or "negative heel" or "negative heels")) or (rocker? or insole?)).ti,ab. (1507)

14 ((HKAFO? or KAFO? or SCKAFO? or AFO? or GRAFO? or RGO? or SWASH? or DAFO? or SAFO?) and (orthos* or orthot* or brace? or bracing)).ti,ab. (387)

15 (SMART? and walker).ti,ab. (10)

16 11 or 12 or 13 or 14 or 15 (5269)

17 10 or 16 (6735)

18 exp Knee Joint/ or Knee/ (51355)

19 knee?.af. (114312)

20 18 or 19 (115529)

21 17 and 20 (2085)

For peer review only

For peer review only

SUPPLEMENTARY FILE 2 QUALITY ASSESSMENT

Randomised controlled trials

Study	Selection bias Random sequence generation	Selection bias Allocation concealment	Performance bias Blinding of participants and personnel	Detection bias Blinding of healthcare professional assessed outcomes	Attrition bias	Selective outcome reporting	Other
Yang [23]	Unclear risk	Unclear risk	High risk<br><br>Not possible due to nature of intervention	High risk of bias<br>Treating clinician assessed outcome which is likely to be influenced by lack of blinding	Low risk of bias	Unclear	
Harvey[24]	Unclear risk	Unclear risk	High risk<br><br>Not possible due to nature of intervention	High risk of bias<br>Treating clinicians appeared to be involved in gathering data on outcomes likely to be influenced by lack of blinding	High risk of bias for ambulatory outcomes	Unclear	Only a small number of patients wore their second device suggesting a cross-over design was not appropriate.

For peer review only - http://bmjopen.bmj.com/site/about/guidelines.xhtml

Non-randomised controlled studies

Study	Selection criteria adequately reported?	Representative sample?	Participation rate ≥80%?	Performance bias?	Independent outcome assessment?	Follow-up ≥80%?	Selection bias?							
							1	2	3	4	5	6	7	8
Morinaka[22]	N	U	N	U	N	NA[a]	Y	Y	U	U	U	U	N[b]	U
Tang[32]	Y	Y	Y	U	N	U	U	U	U	U	U	U	U	U
Whittle[33]	N	U	U	U	N	Y	U	U	U	U	U	U	U	U

1 gender; 2 age; 3 cause of muscle weakness; 4 presence of sensory disturbance; 5 purpose of orthosiss (proximal/distal muscle weakness); 6 previous use of orthosis; 7 acclimatisation time; 8 type of orthosis used

a Appears to be retrospective

b The average time post-stroke was 20 months for the KAFO group and 40 months for the AFO group suggesting likely differences in functioning and time using an orthotic device

For peer review only

Case series

Study	Selection criteria adequately reported?	Representative sample?	Participation rate ≥80%?	Prospective?	Independent outcome assessment?	Follow-up ≥80%?	Prognostic variables reported?	Co-interventions?	Measure of variability?	Other important limitations
Bernhardt[19]	N	U	U	Y	N	N	Y	N	P	Reporting of results
Bocker[10]	Y	Y	U	Y	N	N	N	Y	Y	Reporting of results
Boudarham[20]	Y	U	U	Y	N	U	Y	N	Y	
Brehm[12]	Y	U	U	Y	N	Y	Y	N	P	Reporting of results
Davis[13]	Y	U	U	Y	N	Y	Y	Y	Y	Generalisability of assessing two different modes of using orthosis in clinic
Hachisuka[14]	U	Y	U	Y	N	N	Y	Y	P	Reporting of results
Heim[16]	N	U	U	Y	N	Y	N	N	N	Reporting of results
Jaspers[27]	N	U	N	N	Y	N	Y	Y	NA	
Kakurai[21]	N	U	U	Y	N	Y	Y	N	Y	Ability to actively control knee a confounder for KAFO and AFO comparisons
Middleton[28]	Y	U	U	U	N	Y[a]	Y	Y	Y	Only patients who had successfully completed gait training and

For peer review only

										continued to used the orthosis were administered a questionnaire
Peethambaran[17]	Y	U	U	Y	N	Y	Y	N	Y	Generalisability due to small sample
Scivoletto	U	U	U	Y	N	Y[a]	Y	N	Y	
Steinfeldt[18]	N	U	U	N	N	Y	N	N	N	
Summers[30]	Y	U	U	N	N	NA[b]	N	Y	NA	Lack of information on interview questionnaire
Sun[31]	N	U	U	U	N	N	N	U	N	
Wu[34]	Y	U	U	U	N	Y	Y	Y	Y	Generalisability due to small sample

N, no; NA, not applicable; P, partial; Y, yes; U, unclear

a For outcome/s included in review; b Retrospective study

For peer review only

For peer review only

PRISMA 2009 Checklist

Section/topic	#	Checklist item	Reported on page #
**TITLE**			
Title	1	Identify the report as a systematic review, meta-analysis, or both.	1
**ABSTRACT**			
Structured summary	2	Provide a structured summary including, as applicable: background; objectives; data sources; study eligibility criteria, participants, and interventions; study appraisal and synthesis methods; results; limitations; conclusions and implications of key findings; systematic review registration number.	2
**INTRODUCTION**			
Rationale	3	Describe the rationale for the review in the context of what is already known.	4-5
Objectives	4	Provide an explicit statement of questions being addressed with reference to participants, interventions, comparisons, outcomes, and study design (PICOS).	5
**METHODS**			
Protocol and registration	5	Indicate if a review protocol exists, if and where it can be accessed (e.g., Web address), and, if available, provide registration information including registration number.	7
Eligibility criteria	6	Specify study characteristics (e.g., PICOS, length of follow-up) and report characteristics (e.g., years considered, language, publication status) used as criteria for eligibility, giving rationale.	5
Information sources	7	Describe all information sources (e.g., databases with dates of coverage, contact with study authors to identify additional studies) in the search and date last searched.	5
Search	8	Present full electronic search strategy for at least one database, including any limits used, such that it could be repeated.	Appendix 1
Study selection	9	State the process for selecting studies (i.e., screening, eligibility, included in systematic review, and, if applicable, included in the meta-analysis).	6
Data collection process	10	Describe method of data extraction from reports (e.g., piloted forms, independently, in duplicate) and any processes for obtaining and confirming data from investigators.	7
Data items	11	List and define all variables for which data were sought (e.g., PICOS, funding sources) and any assumptions and simplifications made.	6
Risk of bias in individual studies	12	Describe methods used for assessing risk of bias of individual studies (including specification of whether this was done at the study or outcome level), and how this information is to be used in any data synthesis.	7
Summary measures	13	State the principal summary measures (e.g., risk ratio, difference in means).	6
Synthesis of results	14	Describe the methods of handling data and combining results of studies, if done, including measures of consistency (e.g., I^2 for each meta-analysis.	N/A

For peer review only

PRISMA 2009 Checklist

Section/topic	#	Checklist item	Reported on page #
Risk of bias across studies	15	Specify any assessment of risk of bias that may affect the cumulative evidence (e.g., publication bias, selective reporting within studies).	N/A
Additional analyses	16	Describe methods of additional analyses (e.g., sensitivity or subgroup analyses, meta-regression), if done, indicating which were pre-specified.	N/A
**RESULTS**			
Study selection	17	Give numbers of studies screened, assessed for eligibility, and included in the review, with reasons for exclusions at each stage, ideally with a flow diagram.	Fig 1
Study characteristics	18	For each study, present characteristics for which data were extracted (e.g., study size, PICOS, follow-up period) and provide the citations.	Table 1
Risk of bias within studies	19	Present data on risk of bias of each study and, if available, any outcome level assessment (see item 12).	Appendix 2
Results of individual studies	20	For all outcomes considered (benefits or harms), present, for each study: (a) simple summary data for each intervention group (b) effect estimates and confidence intervals, ideally with a forest plot.	Not possible due to poor reporting
Synthesis of results	21	Present results of each meta-analysis done, including confidence intervals and measures of consistency.	Not possible
Risk of bias across studies	22	Present results of any assessment of risk of bias across studies (see Item 15).	Not possible
Additional analysis	23	Give results of additional analyses, if done (e.g., sensitivity or subgroup analyses, meta-regression [see Item 16]).	Narrative synthesis p7-16
**DISCUSSION**			
Summary of evidence	24	Summarize the main findings including the strength of evidence for each main outcome; consider their relevance to key groups (e.g., healthcare providers, users, and policy makers).	16
Limitations	25	Discuss limitations at study and outcome level (e.g., risk of bias), and at review-level (e.g., incomplete retrieval of identified research, reporting bias).	17
Conclusions	26	Provide a general interpretation of the results in the context of other evidence, and implications for future research.	17-18

For peer review only

For peer review only - http://bmjopen.bmj.com/site/about/guidelines.xhtml

PRISMA 2009 Checklist

**FUNDING**			
Funding	27	Describe sources of funding for the systematic review and other support (e.g., supply of data); role of funders for the systematic review.	19

From: Moher D, Liberati A, Tetzlaff J, Altman DG, The PRISMA Group (2009). Preferred Reporting Items for Systematic Reviews and Meta-Analyses: The PRISMA Statement. PLoS Med 6(7): e1000097. doi:10.1371/journal.pmed1000097

For more information, visit: **www.prisma-statement.org**.

For peer review only

For peer review only - http://bmjopen.bmj.com/site/about/guidelines.xhtml

BMJ Open

A SYSTEMATIC REVIEW OF THE EVIDENCE ON ORTHOTIC DEVICES FOR THE MANAGEMENT OF KNEE INSTABILITY RELATED TO NEUROMUSCULAR AND CENTRAL NERVOUS SYSTEM DISORDERS

Journal:	*BMJ Open*
Manuscript ID	bmjopen-2017-015927.R1
Article Type:	Research
Date Submitted by the Author:	19-Jul-2017
Complete List of Authors:	McDaid, Catriona; University of York, Department of Health Sciences<br>Fayter, Debra; University of York, Centre for Reviews and Dissemination; Kleijnen Systematic Reviews Ltd<br>Booth, Alison; York University, Department of Health Sciences<br>O'Connor, Joanne; University of York, Centre for Reviews and Dissemination<br>Rodriguez-Lopez, Rocio; University of Leeds, Academic Unit of Health Economics<br>Mccaughan, Dorothy; University of York, Department of Health Sciences<br>Bowers, RJ; University of Strathclyde<br>Iglesias, C; University of York, Department of Health Sciences<br>Lalor, Simon; Queen Mary's Hospital<br>O'Connor, Rory J.; University of Leeds<br>Phillips, Margaret Philip; Royal Derby Hospital<br>Ramdharry, Gita; Kingston University; St George's University of London
**Primary Subject Heading**:	Rehabilitation medicine
Secondary Subject Heading:	Evidence based practice
Keywords:	systematic review, orthotic device, knee instability, central nervous system disorder, Neuromuscular disease < NEUROLOGY

SCHOLARONE™ Manuscripts

A SYSTEMATIC REVIEW OF THE EVIDENCE ON ORTHOTIC DEVICES FOR THE MANAGEMENT OF KNEE INSTABILITY RELATED TO NEUROMUSCULAR AND CENTRAL NERVOUS SYSTEM DISORDERS

Catriona McDaid[1], Debra Fayter[2], Alison Booth[1], Joanne O'Connor[2], Rocio Rodriguez-Lopez[3], Dorothy McCaughan[1], Roy Bowers[4], Cynthia P Iglesias[1], Simon Lalor[5], Rory J O'Connor[6], Margaret Phillips[7], Gita Ramdharry[8]

[1]Department of Health Sciences, University of York, York, UK

[2]Centre for Reviews & Dissemination, University of York, York, UK

[3]Academic Unit of Health Economics, University of Leeds, Leeds, UK

[4]Biomedical Engineering, University of Strathclyde, Glasgow, UK

[5]Queen Mary's Hospital, St George's University Hospitals NHS Foundation Trust, London, UK

[6]Leeds Institute of Rheumatic and Musculoskeletal Medicine, University of Leeds, Leeds, UK

[7]Royal Derby Hospital, Derby, UK

[8]Kingston University and St George's University of London, London, UK

Correspondence to:

Dr Catriona McDaid, York Trials Unit, Dept. of Health Sciences, ARRC Building, University of York, York, YO10 5DD; catriona.mcdaid@york.ac.uk

Word Count: 3905

Keywords: systematic review, orthotic devices, knee instability, central nervous system disorder, neuromuscular disease

For peer review only - http://bmjopen.bmj.com/site/about/guidelines.xhtml

ABSTRACT

Objectives: To assess the effectiveness of orthotic devices for the management of instability of the knee in adults with a neuromuscular or central nervous system disorder

Design: A systematic review of primary studies

Setting: Community

Participants: Adults with a neuromuscular or central nervous system disorder and impaired walking ability due to instability of the knee

Interventions: Orthoses with the clinical aim of controlling knee instability for example, knee-ankle-foot orthoses (KAFO), ankle-foot orthoses (AFO) and knee orthoses (KO) or mixed design with no restrictions in design or material.

Primary and secondary outcome measures: condition specific or generic patient-reported outcome measures assessing function, disability, independence, activities of daily living, quality of life or psychosocial outcomes; pain; walking ability; functional assessments; biomechanical analysis; adverse effects; usage; patient satisfaction and the acceptability of a device; and resource utilisation data.

Results: Twenty-one studies including 478 patients were included. Orthotic devices were evaluated in patients who were post-polio, post-stroke, with inclusion body myositis and with spinal cord injury. The review included two randomised controlled trials, three non-randomised controlled studies and 16 case series. Most were small, single centre studies with only six of 21 following patients for one year or longer. They met between one and five of nine quality criteria and reported methods and results poorly. They mainly assessed outcomes related to gait analysis and energy consumption with limited use of standardised, validated patient-reported outcome measures. There was an absence of evidence on outcomes of direct importance to patients such as reduction in pain and falls.

Conclusions: There is a need for high quality research, particularly RCTs, of orthotic devices for knee instability related to neuromuscular and central nervous system conditions. This research should address outcomes important to patients. There may also be value in developing a national registry.

Registration number systematic review: PROSPERO (CRD42014010180).

For peer review only

ARTICLE SUMMARY

Strengths and limitations of this study

- The first systematic review addressing this question to systematically consider study quality.
- An extensive range of sources were searched to identify studies.
- It was difficult to be certain that knee instability was the main problem being treated in some of the studies.
- Due to poor reporting of the primary studies, it was not possible to extract outcome data in the standardised way planned in the protocol.

For peer review only

For peer review only - http://bmjopen.bmj.com/site/about/guidelines.xhtml

INTRODUCTION

Instability can occur in any of the three anatomical planes of the knee: sagittal, coronal or transverse planes and there are several mechanisms that may lead to knee instability in neuromuscular disorders (NMD) and central nervous system (CNS) conditions. These include: weakness or over-activity of any of the muscles that have a direct effect on the knee (knee extensors, knee flexors, gastrocnemius); and muscle weakness or over-activity remote from the muscles directly affecting the knee due to secondary effects on posture (e.g. alterations to the anterior progression of the ground reaction force under the foot, or plantarflexor weakness leading to uncontrolled dorsiflexion). In the case of CNS conditions, spasticity in the muscles around the knee can also cause knee instability (e.g. spasticity in the gastrocnemius causes excessive plantarflexion in stance which shifts the ground reaction force anterior to the knee causing hyperextension).[1] Knee instability can lead to pain, falls and a range of mobility issues for the individual.

Knee instability due to muscle weakness or ligamentous laxity is often treated using orthoses with the functional goals of improving walking and to protect, stabilise and improve function.[2] Knee orthoses (KO) are often prescribed or in some cases a type of ankle-foot orthosis (AFO) known as a Ground Reaction AFO (GRAFO) may be provided. A GRAFO provides direct control of the ankle and foot, and indirect control of the knee and hip may be provided through optimising and normalising the alignment of the Ground Reaction Force in relation to the knee joint throughout stance phase. A knee-ankle-foot orthosis (KAFO) is usually prescribed when bracing with an AFO or KO is insufficient to adequately control knee instability and usually when control in more than one plane is required.[2] Modern KAFOs tend to combine plastic and metal components: commonly polypropylene for calf and thigh shells and shoe inserts, aluminium, magnesium, titanium or steel for uprights and steel for joints.[3] Variations exist in the orthotic knee joint design, locking and unlocking mechanism, type of knee pads and plane of control.[3] A locked knee KAFO requires an altered gait to allow the individual's foot to clear the ground in the swing phase of walking. Polycentric knee joints can be locked or unlocked and permit a more anatomical or natural knee motion, though have more two-joint axes and may require more maintenance and are therefore more expensive.[3] Stance control knee joints have either a mechanical or microprocessor controlled knee joint which allows the knee to flex during the swing phase of walking, but lock during the stance phase of walking, when the knee is extended, allowing a more normal walking pattern. Other, more extensive options include hip-knee-ankle-foot orthoses (HKAFO) originally designed for patients with higher level spinal cord dysfunction who might otherwise have been

For peer review only - http://bmjopen.bmj.com/site/about/guidelines.xhtml

unable to walk.[4] These include hip guidance orthoses (HGOs) and reciprocating gait orthoses (RGOs) which have different locking mechanisms.

We undertook a systematic review with the aim of assessing the evidence base for the effectiveness of orthotic devices for management of instability of the knee in adults who have NMD or a CNS disorder. This was part of a larger mixed-methods project undertaken to inform the development of a future substantive research question on the clinical and cost-effectiveness of different types of orthotic management of the knee in people with NMD or CNS disorders.[1]

METHODS

We undertook searches to identify studies assessing the effectiveness of orthotic devices for management of instability of the knee in adults who have NMD or a CNS disorder.

Search methods for identification of studies

We searched the following databases from inception to November 2014: MEDLINE via Ovid, MEDLINE In-Process via Ovid, Cumulative Index to Nursing & Allied Health (CINAHL) via EBSCO, EMBASE via Ovid, PASCAL via Ebsco, Scopus, Science Citation Index (ISI Web of Knowledge), BIOSIS Previews, PEDro, Recal Legacy, Cochrane Database of Systematic Reviews (CDSR), Database of Abstracts of Reviews of Effects (DARE), Health Technology Assessment (HTA) database and Cochrane Central Register of Controlled Trials (CENTRAL) in The Cochrane Library , Conference Proceedings Citation Index- Science (CPCI-S) (ISI Web of Knowledge), Health Management Information Consortium (HMIC) via Ovid, ClinicalTrials.gov, International Clinical Trials Registry Platform (WHO ICRTP) National Technical Information Service (NTIS) and selected websites. There were no language or publication status restrictions. See online supplementary appendix 1 for the MEDLINE (Ovid) search strategy which was adapted for the other databases.

The reference lists of all included studies, any related systematic reviews and key background papers were checked to identify any further, relevant studies.

Eligibility criteria

Population: Adults (16 years or older) with a neuromuscular or central nervous system disorder and impaired walking ability due to instability of the knee were eligible for inclusion. Children were excluded.

For peer review only - http://bmjopen.bmj.com/site/about/guidelines.xhtml

Intervention: Orthoses with the clinical aim of controlling knee instability for example, knee-ankle-foot orthoses (KAFO), ankle-foot orthoses (AFO) and knee orthoses (KO) or of mixed design with no restrictions in design, material, custom or pre-fabricated; type of knee joint or stance-control design (KAFO), or whether there was an electronic component. Studies evaluating the use of functional electrical stimulation were excluded.

Studies were eligible provided the orthosis had been used in a real-life setting (i.e. studies where the device had been solely used within a laboratory / experimental setting were excluded). Outcomes could be assessed in a laboratory or clinic setting provided participants had used the device in the community.

Comparator: Studies using any of the above orthoses as a comparator; including studies comparing different designs of the same orthosis; or no intervention.

Study design: Randomised controlled trials (RCTs) and other study designs with and without a comparator group such as non-randomised controlled studies, before and after studies and case series were eligible for the review.

The following outcomes were of interest: Condition specific or generic patient-reported outcomes measures assessing function, disability, independence, activities of daily living, quality of life or psychosocial outcomes; pain; walking ability; functional assessments; biomechanical analysis; adverse effects; usage; patient satisfaction and the acceptability of a device; and resource utilisation data.

Two researchers independently screened titles and abstracts and full papers to assess eligibility. Disagreements were resolved through discussion and consultation with a third member of the project team if necessary. Authors were contacted if eligibility was uncertain from the information provided in the publication. There were no language restrictions.

Data extraction

Data were extracted by one researcher and checked by a second with discrepancies resolved by discussion. Studies in languages other than English were extracted by a native speaker who was also a researcher and were checked by a second researcher for consistency only. Data were extracted using a piloted data extraction form. Multiple publications of the same study (linked papers) were extracted and reported as a single study. Between-group differences were extracted from studies with a comparator. We had planned to extract data to allow calculation of between group differences and confidence intervals. However due to the generally poor reporting of data, it was not possible to consistently do this across studies. Where data were available, these were extracted;

For peer review only - http://bmjopen.bmj.com/site/about/guidelines.xhtml

where the appropriate data were not reported, the description of the results provided in the paper was extracted and the lack of summary data noted.

Study quality

RCTs were assessed using the Cochrane risk of bias criteria.[5] Non-randomised studies with a control group were assessed for external validity, performance bias, detection bias and selection bias/control of confounding based on eight criteria (gender, age, cause of muscle weakness, presence of sensory disturbance, whether the orthosis was used for proximal or distal muscle weakness, previous use of an orthosis, acclimatisation time and type of orthosis used). Case series were assessed using criteria adapted from the assessment of controlled studies and criteria used in a previous systematic review.[6] Assessment of risk of bias was undertaken independently by two researchers (except for non-English language studies). Discrepancies were resolved by discussion.

The protocol was registered with PROSPERO in advance of undertaking the review (registration number CRD42014010180). Ethical approval was not required.

RESULTS

Overview of the evidence

A total of 4516 references were identified from the searches and 21 studies of 478 patients (reported in 25 publications) were included (Figure 1). A full list of papers and reasons for exclusion is available from the authors. A substantial proportion were excluded (n=76) because the orthosis was evaluated in a laboratory or clinical setting without the participant using the device in the community. Three potentially relevant ongoing studies were identified: a before and after study;[7] a case series,[8] and an RCT.[9]

Table 1 provides a summary of study characteristics grouped by the four conditions covered by the included studies: post-polio, inclusion body myositis, post stroke, and spinal cord injury. Two RCTs, three non-randomised studies with a control group, and 16 case series were included. Sample sizes ranged from five to 67 participants and just fewer than half the studies had over 20 participants. The follow-up time was generally short, only 6 studies followed patients for one year or longer.

For peer review only - http://bmjopen.bmj.com/site/about/guidelines.xhtml

Table 1: Study characteristics by condition

Main publication (*associated papers*) Country	Study design N in study (n in analyses)	Population % male Mean age (SD)	Intervention (I) Comparator (C)	Co-interventions	Length of follow-up
			**POST-POLIO SYNDROME**		
Bocker, 2013[10] (*Bocker, 2011[11]*) Germany	Case series N=10 (6)	30% 64.5	I: Carbon fibre KAFO C: No comparator	Gait training, pain therapy and exercises (twice per week for 3 months)	3 months
Brehm, 2007[12] Netherlands	Case series N=23 (20)	61% 55 (9.2)	I: Carbon fibre KAFO (locked knee-joint) C: Leather /metal or plastic /metal KAFO used previously by same participants	Walking aids were used by some participants	26 weeks
Davis, 2010[13] Australia	Case series N=10 (10)	40% 61.9 (7.7)	I: Carbon fibre SCKAFO in stance control mode C: KAFO in locked knee mode used by same participants	Walking aids	Mean duration of use at time of evaluation 6.2 (SD 5.2) months
Hachisuka, 2006[14] Japan (*Hachisuka, 2007 [15]*)	Case series N=11 (8 to 11[a])	18% 53.9 (9.8)	I: Carbon fibre KAFO C: Traditional non-carbon KAFO used by same participants	Walking aids	Not reported
Heim, 1997[16] Israel	Case series N=30 (27)	33% 44	I: Carbon fibre KAFO C: No comparator	Not reported	30 months
Peethambaran, 2000[17] USA	Case series N=5 (5)	40% 61.4 (12.4)	I: Carbon titanium KAFO (anterior approach design) C: Plastic KAFO (posterior approach design) used previously by the same participants	Not reported	6 weeks

For peer review only - http://bmjopen.bmj.com/site/about/guidelines.xhtml

Main publication (*associated papers*) Country	Study design N in study (n in analyses)	Population % male Mean age (SD)	Intervention (I) Comparator (C)	Co-interventions	Length of follow-up
Steinfeldt, 2003[18] Germany	Case series N=55 (55)	44% 58	I: Carbon fibre KAFO C: No comparator	Not reported	>3 months
			**INCLUSION BODY MYOSITIS**		
Bernhardt, 2011[19] USA	Case series N=9 (6)	78% 61 (9)	I: SCKAFO C: No comparator	Not reported	6 months
			**POST-STROKE**		
Boudharam, 2013[20] France	Case series N=11 (unclear)	64% 51 (15)	I: Carbon fibre KAFO C: No comparator	Not reported	Device prescribed within past 6 months
Kakurai, 1996[21] Japan	Case series N=28 (28)	50% 54.5	I: Plastic convertible KAFO (to AFO) C: Participants who changed to AFO compared with those remaining on KAFO	Not reported	Not reported
Morinaka, 1982[22] Japan	Cohort study N=25 (25)	64% 56	I: Plastic KAFO C: 50 participants fitted with AFOs and a group of 30 healthy adult males	Not reported	Mean 14.6 months (range 1 to 35)
Yang , 2005[23] China	RCT N=67 (67)	84% 58	I: KAFO or AFO C: 'Conventional rehabilitation'	Not reported	Not reported

For peer review only - http://bmjopen.bmj.com/site/about/guidelines.xhtml

Main publication (*associated papers*) Country	Study design N in study (n in analyses)	Population % male Mean age (SD)	Intervention (I) Comparator (C)	Co-interventions	Length of follow-up
			**SPINAL CORD INJURY**		
Harvey, 1997[24] Australia (*Harvey, 1997;*[25] *Harvey, 1998* [26])	RCT (cross-over) N=10 (5-10[b])	90% 37 (8.4)	I: HKAFO (Walkabout Orthosis) C: HKAFO (IRGO)	Gait training (30 to 54 hours per orthosis) Crutches	28 weeks
Jaspers, 1997[27] Belgium	Case series N=14 (14)	86% 33.6	I: HKAFO (ARGO) C: No comparator	Walker or Crutches	1 year
Middleton, 1997[28] Australia	Case series N=25 (21)	76% 35 (13)	I: HKAFO (Walkabout Orthosis) C: No comparator	Parallel bars, forearm crutches or frames	≥ 18 months
Scivoletto, 2000[29] Italy	Case series N=24 (24[c])	79% 33.6 (3.2)	I: HKAFO (RGO) C: No comparator (internal comparison of non-users versus users)	Not reported	1 year
Summers, 1988[30] UK	Case series N=20 (20)	100% 28	I: HKAFO (HGO ParaWalker) C: No comparator	Crutches used as decided by patient	Mean 20 months
Sun, 2007[31] China	Case series N=20 (15)	67% 33.7	I: HKAFO (RGO) C: No comparator	Not reported	Not reported

For peer review only - http://bmjopen.bmj.com/site/about/guidelines.xhtml

Main publication (*associated papers*) Country	Study design N in study (n in analyses)	Population % male Mean age (SD)	Intervention (I) Comparator (C)	Co-interventions	Length of follow-up
Tang, 2009[32] China	Controlled study N=58 (unclear)	83% 32.4	I: AGO, RGO, KAFO C: Rehabilitation training	Rehabilitation training	4 months[d]
Whittle, 1991[33] UK	Controlled study (cross-over) N=22 (Unclear[e])	82% 34	I: HKAFO (HGO ParaWalker) C: HKAFO (RGO)	Rollator or crutches	4 months
Wu, 2003[34] China	Case series N=6 (6)	67% 27.6	I: HKAFO (Walkabout Orthosis) C: No comparator group	Gait training including balance plus walking exercises	Unclear

SD, Standard deviation

[a] Eight completed assessment of non-carbon fibre KAFO and walking without an orthosis and 11 completed assessment of carbon-fibre KAFO

[b] Appears to be 22 for analysis of final choice of orthosis, although one left the trial without trying either and three participants tried only one. It was unclear how many participants were included in other analyses

[c] Appears to be 10 for all analyses except for speed of walking on flat surface (n=8) and speed of walking on ramp (n=5)

[d] Eight weeks after fitting of device

[e] A total of 24 for the single outcome eligible for the review, although unclear for other analyses in the study

For peer review only

Study Quality

Both RCTs were assessed as having an unclear or high risk of bias for the majority of items on the Cochrane risk of bias tool (Supplementary file 2). Overall the three non-randomised controlled studies were at risk of selection bias (Supplementary file 2). Ten of the 16 studies without a control group were prospective, three were retrospective and this aspect of the design was unclear for three. Overall the 16 studies met between one and five of the nine quality criteria, only eight adequately described their inclusion criteria and all were considered at risk of selection bias (Supplementary file 2). Poor reporting of study methods and results was a problem across all the study designs. Studies often made statements in the results section which were not backed up with numerical data. Where data were available, these were extracted; where the appropriate data were not reported, the description of the results provided in the paper was extracted and the lack of summary data noted in the data extraction tables which are available in the HTA report.[1]

Outcomes

The most systematically assessed outcomes in the included studies were gait quality and energy consumption, assessed during clinic/laboratory visits (Table 2). While several studies (Table 2) reported patient satisfaction with the device and functionality (e.g. how it impacted sitting in their wheelchair; the main ways in which they used the device), the results were predominantly reported in an anecdotal fashion and it was not possible to assess how robustly the information had been collected. Despite our requirement that participants in studies had used their orthoses outside the clinic only one study used a validated measure (Barthel Index) of patients' ability to manage every day activities of daily living outside the clinic setting;[21] and only two assessed quality of life using a validated measure (Table 2). Generally, adverse effects such as skin damage or falls were not systematically reported. It cannot be inferred that there were few adverse events as authors did not specifically mention that no adverse events were identified.

For peer review only - http://bmjopen.bmj.com/site/about/guidelines.xhtml

For peer review only

Table 2: Outcomes assessed

	Patient-reported outcomes					*Objective assessments*			*Resource utilisation*	
**Study**	**Satisfaction with device**	**Functionality of device**	**Usage of device**	**Quality of life**	**Adverse effects**	**Walking ability**	**Energy consumption**	**Muscle activity**	**Device malfunction**	**Cost**
**POST-POLIO**										
Bocker[10]				✓				✓		
Brehm[12]	✓					✓	✓		✓	
Davis[13]						✓	✓			
Hachisuka[14]	✓					✓	✓		✓	✓
Heim[16]	✓		✓		✓				✓	
Peethambaran[17]	✓	✓			✓					
Steinfeldt[18]	✓	✓							✓	
**INCLUSION BODY MYOSITIS**										
Bernhardt[19]	✓	✓	✓			✓				
**POST STROKE**										
Boudharam[20]						✓				
Kakurai[21]						✓		✓		
Morinaka[22]			✓			✓				
Yang[23]								✓		
**SPINAL CORD INJURY**										
Harvey[24]	✓	✓	✓			✓	✓	✓		
Jaspers[27]	✓	✓	✓		✓				✓	
Middleton[28]	✓	✓	✓						✓	
Scivoletto[29]		✓	✓							
Summers[30]	✓	✓	✓		✓				✓	
Sun[31]		✓			✓	✓				

For peer review only - http://bmjopen.bmj.com/site/about/guidelines.xhtml

Tang[32]		✓	✓				
Whittle[33]	✓	✓	✓	✓		✓	✓
Wu[34]		✓		✓	✓		

For peer review only

For peer review only - http://bmjopen.bmj.com/site/about/guidelines.xhtml

Post-polio patients

Seven case series (n=143 patients) investigated types of carbon fibre KAFO (Table 1). Three compared a new device to the one used previously by participants;[12,14,17] one compared using a device in stance control mode and locked mode (with the aim of replicating a traditional KAFO design);[13] two before and after use of the orthosis;[10,18] and post-intervention only in one study.[16]

Outcomes were sparsely reported. Five of the seven studies reported measures of patient satisfaction, although not in sufficient detail to assess the robustness of the evaluation.[12,14,16-18] Three studies made a formal assessment of walking ability,[12-14] and four assessed either energy consumption or particular muscle activity.[10,12-14] Resource utilisation data was limited to assessment of device malfunction in four studies[12,14,16,17] and cost in one study.[14] Five studies failed to report adverse effects data or to mention that no adverse effects were identified.[10,12-14,18]

Inclusion body myositis

One case series study (n=9 patients) evaluated a stance control KAFO. [19] Gait was assessed in the clinic with and without use of the device following six months of use. A questionnaire was designed by the investigators to elicit patient outcomes but the results were not reported in full. No data were reported on resource utilisation or adverse effects.

Post-stroke patients

Four studies (n=131 patients); one RCT,[23] a cohort study,[22] and two case series[20,21]evaluated KAFOs and/or AFOs used for knee instability. One assessed a single outcome, gait with and without use of a carbon fibre KAFO. [20] Two studies compared a thermoplastic KAFO to an AFO for knee instability: one compared patients who had changed to an AFO with those who continued to use a KAFO,[21] effectively the comparison was between those who had recovered sufficient control of knee activity to switch to an AFO compared to those who had not; the second compared KAFO to AFO and to normal adult gait.[22] The RCT compared use of an AFO or KAFO to what was described as conventional rehabilitation (not reported in detail).[23] The only patient reported outcome assessed was usage.[22] Three studies made a formal assessment of walking ability,[20-22] and two assessed other functional abilities.[21,23] None reported on resource utilisation data or adverse effects.

Patients with spinal cord injury

For peer review only - http://bmjopen.bmj.com/site/about/guidelines.xhtml

Nine studies (n=194 patients); one RCT[24] two controlled trials,[32,33] and six case series,[27-31,34] evaluated HKAFOs. The RCT used a cross-over design to compare Walkabout® Orthosis (WO) (Polymedic, Queensland Australia) to an isocentric® RGO (IRGO) (Center for Orthotics Design, Campbell, CA). There was a two month washout period of no orthoses use; the data were analysed as though from a parallel trial.[24] There were two further studies of WO with no comparator.[28,34] Two investigated a HGO, one with no comparator,[30] and one compared the HGO to a custom made RGO worn by the same patients in a crossover study.[33] The remaining four studies investigated types of RGO, two with no comparator,[27,31] one in comparison with RGO non-users,[29] and one compared three different types of orthoses (plus rehabilitation training) to rehabilitation training alone.[32] Although each of the studies reported at least one patient-reported outcome, only one study reported using a validated scale (Barthel Index and Functional Independence Measure); and due to lack of clarity in the analysis and reporting it is unclear whether there were any between group differences at follow-up in this study. [32] There were fewer objective assessments across the studies than for the other conditions.[24,31,33,34] Resource utilisation data was limited to assessment of device malfunction [27,28,30,33] and cost.[33] Two thirds of studies did not address adverse effects.[24,28,29,32-34]

DISCUSSION

Principal findings

The review identified a paucity of high quality evidence assessing the effectiveness of orthotic devices for knee instability experienced by people with neuromuscular and central nervous system conditions. In addition to the very limited use of robust study designs, in particular RCTs, reporting was generally poor. For example several studies made statements about findings without presentation of supporting data. The evidence base consists of small, single centre studies with outcome assessments that did not appear to have been undertaken independently of treating clinicians. Laboratory-based studies can provide useful insights about efficacy, particularly during development of a device. However the literature is dominated by laboratory evaluations of orthoses: 76 studies were excluded because the evaluation of the orthosis did not include any use of the device by the patient in a non-clinic setting and the most systematically assessed outcomes in the included studies focused on gait analysis and energy consumption. There was limited use of standardised, validated patient reported outcome measures. In particular there was an absence of evidence on outcomes that are reported by patients to be important to them such as reduction in pain, falls or trips, improved balance and stability and participation in paid employment, outdoor

For peer review only - http://bmjopen.bmj.com/site/about/guidelines.xhtml

activities (such as gardening), family visits and social events. [1] In addition, fewer than one third of the studies followed patients for a year or more. It is unlikely that studies of less than one year duration fully capture the effects of using the devices.

Given that patients report that orthotic devices prescribed for knee instability can play a crucial role in maintaining, promoting and enhancing physical and psychological health and well-being and participation in employment, family and social community activities, [1] the evidence gaps identified by our review are significant and important. A factor that might contribute to this discrepancy in outcome measurement is current requirements for device regulation; only evidence of performance and safety is required for medical devices associated with lower levels of risk to patients such as orthotics for knee instability. This may result in a lack of incentives to conduct primary research on efficacy and/or effectiveness.[35]

Strengths and weaknesses of the study

We undertook systematic searches across an extensive range of sources for published, unpublished and ongoing studies. There were no language restrictions and we included three studies published in Chinese[23,31,32] and one in German.[18] We assessed the risk of bias in the included studies and used standard methods to reduce error and bias at key stages of the review process. Several studies provided a descriptive report of some outcomes with no numerical data. Due to the paucity of evidence we extracted these reports in order to provide as clear a picture as possible of what information is currently available. Arguably this overestimates the amount of evidence that is available.

During study selection it was often difficult to definitively determine whether the participants had knee instability. This was partly due to poor reporting and partly because knee instability was sometimes part of a more complex problem with stability and mobility and is not an explicit and well defined clinical diagnosis. As a result studies may have been included where it is arguable whether knee instability was the main problem and studies rejected that arguably do include people with knee instability. However, we would not expect that this would in any way change the overall conclusions of the review about the lack of high quality evidence or allow conclusions to be made about the effectiveness of specific devices.

An evidence base of small single-centre studies and inadequate study design is similar to that identified in other reviews of orthotic devices for different populations.[36] [37] [38] Also a systematic review of questionnaires used to assess patient satisfaction with orthoses for any limb

For peer review only - http://bmjopen.bmj.com/site/about/guidelines.xhtml

found that 63% of the 106 included papers used questionnaires developed for the specific study rather than validated measures, supporting our findings on this aspect of the evidence.[39]

Unanswered questions and future research

There is a large gap in the evidence on the effectiveness of KAFOs, AFOs and other orthotic devices for managing knee instability related to NMD and CNS conditions. Robust research is required addressing outcomes that are important to patients. RCTs are the most robust way of assessing effectiveness and a pragmatic trial that recognises that provision of an orthotic device is a complex intervention would be appropriate.

There are a number of challenges for researchers and clinicians to consider when designing future studies, including: defining the target population and knee instability, the personalisation of treatment including customisation of devices, the relative rarity of the problem within individual conditions and whether a trial including patients with knee instability with a range of NMD or CNS conditions would be generalisable. It may also be worth considering a national registry to systematically collect data on the ambulatory problem, devices provided, key elements of management of the instability, factors that inform / determine the process of matching patients to orthotic devices, collection of a core set of standardised and validated patient-reported outcome measures, data on use of the device and resource use. While registries do have limitations this would be a major step change from the current evidence base in terms of increased rigour and generalisability and would create a population database and an infrastructure from which future RCTs could be undertaken. The evidence base in this field could also be improved through systematic development of a core set of outcome measures (http://www.comet-initiative.org/). Future research regardless of study design should follow reporting standards (http://www.equator-network.org/).

CONCLUSIONS

There is a need for high quality research, in particular RCTs, on the effectiveness of KAFOs, AFOs and other orthotic devices for managing knee instability related to NMD and CNS conditions. This research should address outcomes that are important to patients. There may also be value in developing a national registry.

For peer review only - http://bmjopen.bmj.com/site/about/guidelines.xhtml

COMPETING INTERESTS

During this study Simon Lalor was an employee of Opcare, a company that provides orthotic and prosthetic services to the UK NHS. This company does not manufacture orthotic devices, although a sister company ORTHO C FAB does. Cynthia Iglesias is a member of the National Institute for Health and Care Excellence Medical Technologies Assessment Committee and member of the European Clinical Research Infrastructure Network.

AUTHOR'S CONTRIBUTIONS

CMcD was responsible for writing the protocol and had overall responsibility for co-ordinating and leading the project, provided advice and input to all elements of the project and contributed to report writing. AB, DF, JO'C undertook study selection, quality assessment, report writing and contributed to the protocol. RRL provided information specialist support, designed and undertook literature searches and wrote the related sections in the report

RB, CPI, SL, MP, GR, DMcC, and RJO'C were members of the Advisory Group, contributed to the systematic review protocol and/or provided clinical and/or methodological advice throughout the review and commented on drafts of the systematic review report.

CMcD drafted this manuscript and all authors reviewed, edited and approved.

FUNDING STATEMENT

This project was funded by the National Institute for Health Research (NIHR) HTA Programme (project number 13/30/02) and has been published in full in *Health Technol Assess* 2016;20(55). Further information available at https://www.journalslibrary.nihr.ac.uk/programmes/hta/133002/#/

This report presents independent research commissioned by the NIHR. The views and opinions expressed by authors in this publication are those of the authors and do not necessarily reflect those of the NHS, the NIHR, MRC, CCF, NETSCC, the HTA programme or the Department of Health.

DATA SHARING STATEMENT

Most of the data are available in the main body and appendices of the HTA Monograph https://www.journalslibrary.nihr.ac.uk/hta/hta20550/#/abstract
Any further data can be obtained from the corresponding author.

For peer review only - http://bmjopen.bmj.com/site/about/guidelines.xhtml

REFERENCES

1. O'Connor J, McCaughan D, McDaid C, et al. Orthotic management of instability of the knee related to neuromuscular and central nervous system disorders: systematic review, qualitative study, survey and costing analysis. Health Technol Assess 2016;**20**(55)
2. Hebert JS. Ambulatory KAFOs: a physiatry perspective. Journal of Prosthetics & Orthotics 2006;**74**(30 ref)
3. Edelstein J, Bruckner J. *Orthotics: A Comprhensive Clinical Approach*. New Jersey, USA: Slack Incorporated, 2002.
4. Campbell JH. Linked hip-knee-ankle-foot orthoses designed for reciprocal gait. Journal of Prosthetics and Orthotics 2006;**18**(7 PROCEEDINGS):P204-P08
5. Higgins J, Altman D, Sterne J. Chapter 8:Assessing risk of bias in included studies. In: Higgins J, Grees S, eds. Cochrane Handbook for Systematic Reviews of Interventions Version 5.1.0 (updated March 2011): The Cochrane Collaboration, 2011.
6. Llewellyn A, Norman G, Harden M, et al. Interventions for adult Eustachian tube dysfunction: a systematic review. Health Technol Assess 2014;**18**(46)
7. ClinicalTrials.gov. The Use of Brace to Retrain Hemiparetic Gait. URL: http://ClinicalTrials.gov/show/NCT02082938 (accessed 23 May 2016).
8. Kannenberg A, Probsting E. An orthotronic mobility system improves perceived walking capabilities in traditional leg orthosis users. PM and R 2014;**1)**:S229-S30
9. Frechtel A, Portnoy S, Raveh E, et al. Prevention of knee hyperextension in stroke patients using a knee orthosis: 3D computational gait analysis and dynamic EMG. Gait Posture 2013;**38**:S85. http://dx.doi.org/10.1016/j.gaitpost.2013.07.178
10. Bocker B, Hoelig C, Smolenski UC. Orthosis Management in Patients after Poliomyelitis Anterior Acuta. Physikalische Medizin Rehabilitationsmedizin Kurortmedizin 2013;**23**(1):16-21 doi: 10.1055/s-0032-1331218[published Online First: Epub Date]|.
11. Bocker B, Hölig C, Smolenski UC. Orthosis management in patients after poliomyelitis anterior acuta. J Rehabil Med 2011;**49**:40
12. Brehm MA, Beelen A, Doorenbosch CA, et al. Effect of carbon-composite knee-ankle-foot orthoses on walking efficiency and gait in former polio patients. J Rehabil Med 2007;**39**(8):651-7
13. Davis PC, Bach TM, Pereira DM. The effect of stance control orthoses on gait characteristics and energy expenditure in knee-ankle-foot orthosis users. Prosthet Orthot Int 2010;**34**(2):206-15. http://dx.doi.org/10.3109/03093641003773189
14. Hachisuka K, Makino K, Wada F, et al. Clinical application of carbon fibre reinforced plastic leg orthosis for polio survivors and its advantages and disadvantages. Prosthet Orthot Int2006;**30**(2):129-35
15. Hachisuka K, Makino K, Wada F, et al. Oxygen consumption, oxygen cost and physiological cost index in polio survivors: a comparison of walking without orthosis, with an ordinary or a carbon-fibre reinforced plastic knee-ankle-foot orthosis. J Rehabil Med 2007;**39**(8):646-50
16. Heim M, Yaacobi E, Azaria M. A pilot study to determine the efficiency of lightweight carbon fibre orthoses in the management of patients suffering from post-poliomyelitis syndrome. Clin Rehabil 1997;**11**(4):302-5
17. Peethambaran A. The relationship between performance, satisfaction, and well being for patients using anterior and posterior design knee-ankle-foot-orthosis. Journal of Prosthetics and Orthotics 2000;**12**(1):33-40
18. Steinfeldt F, Seifert W, Gunther KP. Modern carbon fibre orthoses in the management of polio patients--a critical evaluation of the functional aspects. Z Orthop Ihre Grenzgeb 2003;**141**(3):357-61
19. Bernhardt K, Oh T, Kaufman K. Stance control orthosis trial in patients with inclusion body myositis. Prosthet Orthot Int 2011;**35**(1):39-44. http://dx.doi.org/10.1177/0309364610389352

For peer review only - http://bmjopen.bmj.com/site/about/guidelines.xhtml

20. Boudarham J, Zory R, Genet F, et al. Effects of a knee-ankle-foot orthosis on gait biomechanical characteristics of paretic and non-paretic limbs in hemiplegic patients with genu recurvatum. Clin Biomech 2013;**28**(1):73-8. http://dx.doi.org/10.1016/j.clinbiomech.2012.09.007
21. Kakurai S, Akai M. Clinical experiences with a convertible thermoplastic knee-ankle-foot orthosis for post-stroke hemiplegic patients. Prosthet Orthot Int 1996;**20**(3):191-4
22. Morinaka Y, Matsuo Y, Nojima M, et al. Clinical evaluation of a knee-ankle-foot-orthosis for hemiplegic patients. Prosthet Orthot Int 1982;**6**(2):111-5
23. Yang JL, Xu YL, Wei Y, et al. Effects of lower limb orthosis therapy on the recovery of motor function in the post-stroke hemiplegic patients. [Chinese]. Chinese Journal of Clinical Rehabilitation 2005;**9**(9):6-7
24. Harvey LA, Newton-John T, Davis GM, et al. A comparison of the attitude of paraplegic individuals to the Walkabout Orthosis and the Isocentric Reciprocal Gait Orthosis. Spinal Cord 1997;**35**(9):580-84
25. Harvey LA, Smith MB, Davis GM, et al. Functional outcomes attained by T9-12 paraplegic patients with the walkabout and the isocentric reciprocal gait orthoses. Arch Phys Med Rehabil 1997;**78**(7):706-11
26. Harvey LA, Davis GM, Smith MB, et al. Energy expenditure during gait using the walkabout and isocentric reciprocal gait orthoses in persons with paraplegia. Arch Phys Med Rehabil 1998;**79**(8):945-9
27. Jaspers P, Peeraer L, Van Petegem W, et al. The use of an advanced reciprocating gait orthosis by paraplegic individuals: a follow-up study. Spinal Cord 1997;**35**(9):585-9
28. Middleton JW, Yeo JD, Blanch L, et al. Clinical evaluation of a new orthosis, the 'walkabout', for restoration of functional standing and short distance mobility in spinal paralysed individuals. Spinal Cord 1997;**35**(9):574-9
29. Scivoletto G, Petrelli A, Lucente LD, et al. One year follow up of spinal cord injury patients using a reciprocating gait orthosis: preliminary report. Spinal Cord 2000;**38**(9):555-8
30. Summers BN, McClelland MR, el Masri WS. A clinical review of the adult hip guidance orthosis (Para Walker) in traumatic paraplegics. Paraplegia 1988;**26**(1):19-26
31. Sun JL, Tang D, Ouyang YT, et al. Influence of reciprocating gait orthosis on walking function in paraplegic patients after ambulation. [Chinese]. Journal of Clinical Rehabilitative Tissue Engineering Research 2007;**11**(13):2437-40
32. Tang D, Pei G, Li K. The effects of alternative gait orthosis on activity of daily living and quality of life in patients with spinal cord injury. Chinese Journal of Rehabilitation Medicine 2009;**24**(11):985-88
33. Whittle MW, Cochrane GM, Chase AP, et al. A comparative trial of two walking systems for paralysed people. Paraplegia 1991;**29**(2):97-102
34. Wu JX, Zhou XL, Liu HL, et al. Effect of the new reciprocating gait orthosis (Walkabout orthosis) in improving paraplegic patients' independent living ability. Chinese Journal of Clinical Rehabilitation 2003;**7**(17):2469-70
35. Iglesias C. Does assessing the value for money of therapeutic medical devices require a flexible approach. Pharmacoeconomics Outcomes Research 2015;**15**(1):21-32
36. Tyson SF, Sadeghi-Demneh E, Nester CJ. A systematic review and meta-analysis of the effect of an ankle-foot orthosis on gait biomechanics after stroke. Clin Rehabil 2013;**27**(10):879-91. http://dx.doi.org/10.1177/0269215513486497
37. Fatone S. A review of the literature pertaining to KAFOs and HKAFOs for ambulation. Journal of Prosthetics and Orthotics 2006;**18**(7 Proceedings):P137-P68
38. Chisholm AE, Perry SD. Ankle-foot orthotic management in neuromuscular disorders: recommendations for future research. Disabil 2012;**7**(6):437-49. http://dx.doi.org/10.3109/17483107.2012.680940

For peer review only - http://bmjopen.bmj.com/site/about/guidelines.xhtml

39. Bettoni E, Ferriero G, Bakhsh H, et al. A systematic review of questionnaires to assess patient satisfaction with limb orthoses. Prosthet Orthot Int 2014;Published Online first:26 November 2014 doi: 10.1177/0309364614556836.

LEGEND

Figure 1 Study Selection

For peer review only

For peer review only - http://bmjopen.bmj.com/site/about/guidelines.xhtml

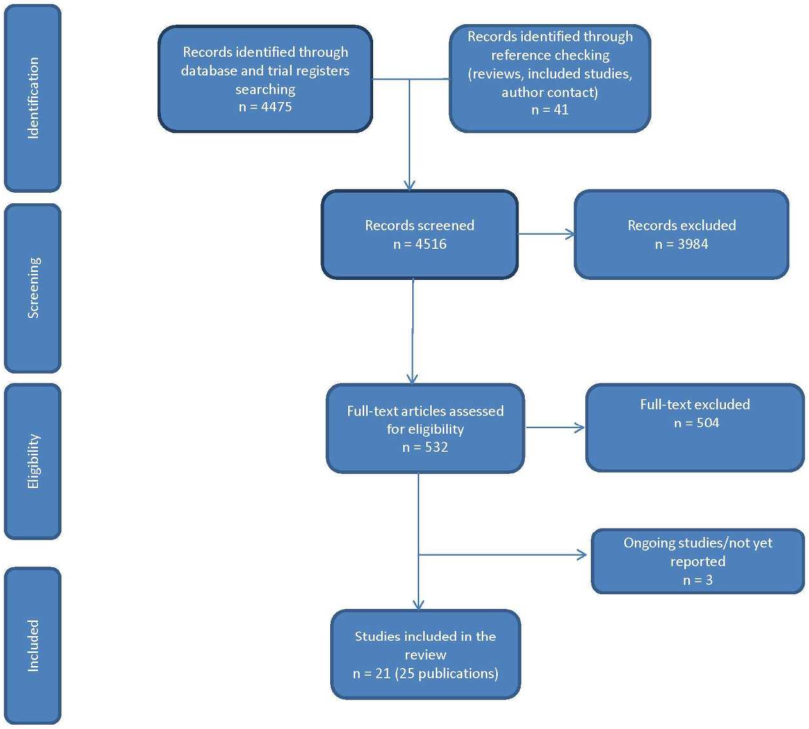

Figure 1

169x127mm (300 x 300 DPI)

ew only

SUPPLEMENTARY APPENDIX 1

Database: Ovid MEDLINE(R) In-Process & Other Non-Indexed Citations and Ovid MEDLINE(R) <1946 to Present> (searched online 22/05/14)

Search Strategy:

1 Orthotic Devices/ or Braces/ or Splints/ (16320)

2 Gait/ (17744)

3 Lower Extremity/ or Leg/ (61929)

4 Hip/ or Hip Joint/ (28943)

5 Knee/ or exp Knee Joint/ (51355)

6 Ankle/ or Ankle Joint/ (16707)

7 Foot/ or Foot Joints/ (20388)

8 1 and (2 or 3 or 4 or 5 or 6 or 7) (2732)

9 Foot Orthoses/ (145)

10 8 or 9 (2870)

11 ((gait or "lower extremity" or "lower extremities" or "lower limb" or "lower limbs" or leg? or hip? or knee? or ankle? or foot or feet) adj3 (orthos* or orthot* or brace? or bracing or support)).ti,ab. (3590)

12 (heel adj2 (pad? or raise?)).ti,ab. (365)

13 ((shoe? and (modification? or insert? or "negative heel" or "negative heels")) or (rocker? or insole?)).ti,ab. (1507)

14 ((HKAFO? or KAFO? or SCKAFO? or AFO? or GRAFO? or RGO? or SWASH? or DAFO? or SAFO?) and (orthos* or orthot* or brace? or bracing)).ti,ab. (387)

15 (SMART? and walker).ti,ab. (10)

16 11 or 12 or 13 or 14 or 15 (5269)

17 10 or 16 (6735)

18 exp Knee Joint/ or Knee/ (51355)

19 knee?.af. (114312)

20 18 or 19 (115529)

21 17 and 20 (2085)

For peer review only

For peer review only

SUPPLEMENTARY FILE 2 QUALITY ASSESSMENT

Randomised controlled trials

Study	Selection bias Random sequence generation	Selection bias Allocation concealment	Performance bias Blinding of participants and personnel	Detection bias Blinding of healthcare professional assessed outcomes	Attrition bias	Selective outcome reporting	Other
Yang [23]	Unclear risk	Unclear risk	High risk<br><br>Not possible due to nature of intervention	High risk of bias<br>Treating clinician assessed outcome which is likely to be influenced by lack of blinding	Low risk of bias	Unclear	
Harvey[24]	Unclear risk	Unclear risk	High risk<br><br>Not possible due to nature of intervention	High risk of bias<br>Treating clinicians appeared to be involved in gathering data on outcomes likely to be influenced by lack of blinding	High risk of bias for ambulatory outcomes	Unclear	Only a small number of patients wore their second device suggesting a cross-over design was not appropriate.

For peer review only

Non-randomised controlled studies

Study	Selection criteria adequately reported?	Representative sample?	Participation rate ≥80%?	Performance bias?	Independent outcome assessment?	Follow-up ≥80%?	Selection bias?							
							1	2	3	4	5	6	7	8
Morinaka[22]	N	U	N	U	N	NA[a]	Y	Y	U	U	U	U	N[b]	U
Tang[32]	Y	Y	Y	U	N	U	U	U	U	U	U	U	U	U
Whittle[33]	N	U	U	U	N	Y	U	U	U	U	U	U	U	U

1 gender; 2 age; 3 cause of muscle weakness; 4 presence of sensory disturbance; 5 purpose of orthosiss (proximal/distal muscle weakness); 6 previous use of orthosis; 7 acclimatisation time; 8 type of orthosis used

a Appears to be retrospective

b The average time post-stroke was 20 months for the KAFO group and 40 months for the AFO group suggesting likely differences in functioning and time using an orthotic device

For peer review only

Case series

Study	Selection criteria adequately reported?	Representative sample?	Participation rate ≥80%?	Prospective?	Independent outcome assessment?	Follow-up ≥80%?	Prognostic variables reported?	Co-interventions?	Measure of variability?	Other important limitations
Bernhardt[19]	N	U	U	Y	N	N	Y	N	P	Reporting of results
Bocker[10]	Y	Y	U	Y	N	N	N	Y	Y	Reporting of results
Boudarham[20]	Y	U	U	Y	N	U	Y	N	Y	
Brehm[12]	Y	U	U	Y	N	Y	Y	N	P	Reporting of results
Davis[13]	Y	U	U	Y	N	Y	Y	Y	Y	Generalisability of assessing two different modes of using orthosis in clinic
Hachisuka[14]	U	Y	U	Y	N	N	Y	Y	P	Reporting of results
Heim[16]	N	U	U	Y	N	Y	N	N	N	Reporting of results
Jaspers[27]	N	U	N	N	Y	N	Y	Y	NA	
Kakurai[21]	N	U	U	Y	N	Y	Y	N	Y	Ability to actively control knee a confounder for KAFO and AFO comparisons
Middleton[28]	Y	U	U	U	N	Y[a]	Y	Y	Y	Only patients who had successfully completed gait training and

										continued to used the orthosis were administered a questionnaire
Peethambaran[17]	Y	U	U	Y	N	Y	Y	N	Y	Generalisability due to small sample
Scivoletto	U	U	U	Y	N	Y[a]	Y	N	Y	
Steinfeldt[18]	N	U	U	N	N	Y	N	N	N	
Summers[30]	Y	U	U	N	N	NA[b]	N	Y	NA	Lack of information on interview questionnaire
Sun[31]	N	U	U	U	N	N	N	U	N	
Wu[34]	Y	U	U	U	N	Y	Y	Y	Y	Generalisability due to small sample

N, no; NA, not applicable; P, partial; Y, yes; U, unclear

a For outcome/s included in review; b Retrospective study

For peer review only

For peer review only

PRISMA 2009 Checklist

Section/topic	#	Checklist item	Reported on page #
**TITLE**			
Title	1	Identify the report as a systematic review, meta-analysis, or both.	1
**ABSTRACT**			
Structured summary	2	Provide a structured summary including, as applicable: background; objectives; data sources; study eligibility criteria, participants, and interventions; study appraisal and synthesis methods; results; limitations; conclusions and implications of key findings; systematic review registration number.	2
**INTRODUCTION**			
Rationale	3	Describe the rationale for the review in the context of what is already known.	4-5
Objectives	4	Provide an explicit statement of questions being addressed with reference to participants, interventions, comparisons, outcomes, and study design (PICOS).	5
**METHODS**			
Protocol and registration	5	Indicate if a review protocol exists, if and where it can be accessed (e.g., Web address), and, if available, provide registration information including registration number.	7
Eligibility criteria	6	Specify study characteristics (e.g., PICOS, length of follow-up) and report characteristics (e.g., years considered, language, publication status) used as criteria for eligibility, giving rationale.	5
Information sources	7	Describe all information sources (e.g., databases with dates of coverage, contact with study authors to identify additional studies) in the search and date last searched.	5
Search	8	Present full electronic search strategy for at least one database, including any limits used, such that it could be repeated.	Appendix 1
Study selection	9	State the process for selecting studies (i.e., screening, eligibility, included in systematic review, and, if applicable, included in the meta-analysis).	6
Data collection process	10	Describe method of data extraction from reports (e.g., piloted forms, independently, in duplicate) and any processes for obtaining and confirming data from investigators.	7
Data items	11	List and define all variables for which data were sought (e.g., PICOS, funding sources) and any assumptions and simplifications made.	6
Risk of bias in individual studies	12	Describe methods used for assessing risk of bias of individual studies (including specification of whether this was done at the study or outcome level), and how this information is to be used in any data synthesis.	7
Summary measures	13	State the principal summary measures (e.g., risk ratio, difference in means).	6
Synthesis of results	14	Describe the methods of handling data and combining results of studies, if done, including measures of consistency (e.g., I^2) for each meta-analysis.	N/A

For peer review only - http://bmjopen.bmj.com/site/about/guidelines.xhtml

PRISMA 2009 Checklist

Section/topic	#	Checklist item	Reported on page #
Risk of bias across studies	15	Specify any assessment of risk of bias that may affect the cumulative evidence (e.g., publication bias, selective reporting within studies).	N/A
Additional analyses	16	Describe methods of additional analyses (e.g., sensitivity or subgroup analyses, meta-regression), if done, indicating which were pre-specified.	N/A
**RESULTS**			
Study selection	17	Give numbers of studies screened, assessed for eligibility, and included in the review, with reasons for exclusions at each stage, ideally with a flow diagram.	Fig 1
Study characteristics	18	For each study, present characteristics for which data were extracted (e.g., study size, PICOS, follow-up period) and provide the citations.	Table 1
Risk of bias within studies	19	Present data on risk of bias of each study and, if available, any outcome level assessment (see item 12).	Appendix 2
Results of individual studies	20	For all outcomes considered (benefits or harms), present, for each study: (a) simple summary data for each intervention group (b) effect estimates and confidence intervals, ideally with a forest plot.	Not possible due to poor reporting
Synthesis of results	21	Present results of each meta-analysis done, including confidence intervals and measures of consistency.	Not possible
Risk of bias across studies	22	Present results of any assessment of risk of bias across studies (see Item 15).	Not possible
Additional analysis	23	Give results of additional analyses, if done (e.g., sensitivity or subgroup analyses, meta-regression [see Item 16]).	Narrative synthesis p7-16
**DISCUSSION**			
Summary of evidence	24	Summarize the main findings including the strength of evidence for each main outcome; consider their relevance to key groups (e.g., healthcare providers, users, and policy makers).	16
Limitations	25	Discuss limitations at study and outcome level (e.g., risk of bias), and at review-level (e.g., incomplete retrieval of identified research, reporting bias).	17
Conclusions	26	Provide a general interpretation of the results in the context of other evidence, and implications for future research.	17-18

For peer review only

For peer review only - http://bmjopen.bmj.com/site/about/guidelines.xhtml

PRISMA 2009 Checklist

FUNDING			
Funding	27	Describe sources of funding for the systematic review and other support (e.g., supply of data); role of funders for the systematic review.	19

From: Moher D, Liberati A, Tetzlaff J, Altman DG, The PRISMA Group (2009). Preferred Reporting Items for Systematic Reviews and Meta-Analyses: The PRISMA Statement. PLoS Med 6(7): e1000097. doi:10.1371/journal.pmed1000097

For more information, visit: **www.prisma-statement.org**.

For peer review only

For peer review only - http://bmjopen.bmj.com/site/about/guidelines.xhtml
